# The MAPK-Signaling Pathway in Colorectal Cancer: Dysregulated Genes and Their Association With MicroRNAs

**DOI:** 10.1177/1176935118766522

**Published:** 2018-03-26

**Authors:** Martha L Slattery, Lila E Mullany, Lori C Sakoda, Roger K Wolff, Wade S Samowitz, Jennifer S Herrick

**Affiliations:** 1 School of Medicine, University of Utah, Salt Lake City, UT, USA; 2 Division of Research, Kaiser Permanente Northern California, Oakland, CA, USA; 3 Department of Pathology, University of Utah, Salt Lake City, UT, USA

**Keywords:** MAPK, colorectal cancer, miRNA, mRNA, ERK1/2

## Abstract

Mitogen-activated protein kinase (MAPK) pathways regulate many cellular functions including cell proliferation and apoptosis. We examined associations of differential gene and microRNA (miRNA) expression between carcinoma and paired normal mucosa for 241 genes in the KEGG-identified MAPK-signaling pathway among 217 colorectal cancer (CRC) cases. Gene expression data (RNA-Seq) and miRNA expression data (Agilent Human miRNA Microarray V19.0; Agilent Technologies Inc., Santa Clara, CA, USA) were analyzed. We first identified genes most strongly associated with CRC using a fold change (FC) of >1.50 or <0.67) that were statistically significant after adjustment for multiple comparisons. We then determined miRNAs associated with dysregulated genes and through miRNA:mRNA (messenger RNA) seed region matches discerned genes with a greater likelihood of having a direct biological association. Ninety-nine genes had a meaningful FC for all CRC, microsatellite unstable–specific tumors, or microsatellite stable–specific tumors. Thirteen dysregulated genes were associated with miRNAs, totaling 68 miRNA:mRNA associations. Thirteen of the miRNA:mRNA associations had seed region matches where the differential expression between the miRNA and mRNA was inversely related suggesting a direct association as a result of their binding. Several direct associations, upstream of ERK1/ERK2, JNK, and p38, were found for *PDGFRA* with 7 miRNAs; *RASGRP3* and *PRKCB* with miR-203a; and *TGFBR1* with miR-6071 and miR-2117. Other associations between miRNAs and mRNAs are most likely indirect, resulting from feedback and feed forward loops. Our results suggest that miRNAs may alter MAPK signaling through direct binding with key genes in this pathway. We encourage others to validate results in targeted CRC experiments that can help solidify important therapeutic targets.

## Introduction

Mitogen-activated protein kinases (MAPKs) are involved in the regulation of cell proliferation, differentiation, migration, and apoptosis and can influence gene expression.^1^ The 3 major MAPK pathways are extracellular-regulated kinases 1 and 2 (ERK1/ERK2), c-Jun-N-terminal kinases (JNKs), and p38 (MAPK14). ERK1/ERK2s are activated by growth factors and cytokines.^1,2^ The JNK pathway is involved in regulating responses to stress, inflammation, and apoptosis and is activated by radiation, environmental stresses, and growth factors. *P38* MAPKs are involved in autoimmunity and are activated by chemical stresses, hormones, and cytokines including IL-1 (interleukin 1) and TNF (tumor necrosis factor)^1,3^ and targets several transcription factors (TFs) including NF-κB (nuclear factor κB) and *TP53*.^3^ Within each of these major MAPK pathways is 3-tiered and includes an MAP kinase kinase kinase (MAP3K, MEKK, or MKKK), MAP kinase kinase (MAP2K, MEK, or MKK), and the MAP kinase (MAPK). Dual-specificity MAPK phosphatases (MKPs or DUSPs) modulate the activity of MAPKs.

MAPK signaling can mediate microRNAs (miRNAs), small noncoding RNAs that regulate gene expression through translation repression or degradation of messenger RNA (mRNAs).^4^ One mechanism through which MAPK may affect miRNAs is through their ability to stabilize Dicer, the enzyme that cleaves double-stranded RNA and pre-miRNA into small-interfering RNA and miRNA. *TRBP* (HIV-1 TAR RNA-binding protein), which is regulated by the MAPK pathway ERK, stabilizes Dicer, regulating miRNA biogenesis.^4,5^ In addition, several miRNAs have been associated with components of MAPK signaling. MiR-101 has been shown to target MAPK phosphatase-1 (MKP-1), which is involved in inflammatory response.^6^ Although miR-101 has been linked to MKP-1, many other miRNAs have been associated with various components of immune response,^6^ suggesting that other miRNAs also might be related to MKP-1. Both miR-4728 and miR-564 have been shown to be an antagonist of MAPK signaling in breast cancer.^7,8^ The interactions between miRNAs and genes in the MAPK-signaling pathway in colorectal cancer (CRC) have not been explored, although studies have shown the importance of MAPK signaling in the cause and progression of CRC.^9^

In this study, we focused on the genes identified in Kyoto Encyclopedia of Genes and Genomes (KEGG) MAPK-signaling pathway. We identified genes within the MAPK-signaling pathway that were significantly dysregulated in CRC tissue when comparing carcinoma with adjacent normal mucosa. Evaluation of those mRNAs with a fold change (FC) of >1.50 or <0.67 with dysregulated miRNAs enabled us to identify miRNA:mRNA associations. Further evaluation of seed region matches between the miRNA and mRNA provided an indication of whether the miRNA was directly influencing gene expression or if the association was one of an indirect nature in that gene expression was altered because of binding elsewhere that influenced feedback loops in the pathway.

## Methods

### Study participants

Two population-based case-control studies, including all incident patients with colon and rectal cancer diagnosed between 30 and 79 years of age in Utah or who were members of Kaiser Permanente Northern California (KPNC), were used. For the colon cancer study, participants were non-Hispanic white, Hispanic, or black; for the rectal cancer study, Asian participants were included.^10,11^ Cases were verified by tumor registry as a first primary adenocarcinoma of the colon or rectum, diagnosed between October 1991 and September 1994 (colon study) or between May 1997 and May 2001 (rectal study).^12^ The study was approved by the Institutional Review Boards at the University of Utah and at KPNC.

### RNA processing

Formalin-fixed paraffin embedded tissue from either the initial biopsy or surgery was used for RNA extraction. RNA was extracted, isolated, and purified from carcinoma tissue and adjacent normal mucosa as previously described.^13^ We observed no differences in RNA quality based on age of the tissue.

### mRNA: RNA-Seq sequencing library preparation and data processing

Total RNA from 245 colorectal carcinoma and normal mucosa pairs was chosen for sequencing based on availability of RNA and high-quality miRNA data to have both mRNA and miRNA from the same individuals; the 217 pairs that passed quality control (QC) were used in these analyses.^14^ RNA library construction was performed with the Illumina TruSeq Stranded Total RNA Sample Preparation Kit with Ribo-Zero (Illumina Inc., San Diego, CA, USA). The samples were then fragmented and primed for complementary DNA (cDNA) synthesis, adapters were then ligated onto the cDNA, and the resulting samples were then amplified using polymerase chain reaction (PCR); the amplified library was then purified using Agencourt AMPure XP beads (Beckman Coulter, Inc. CA, USA). A more detailed description of the methods can be found in our previous work.^15^ Illumina TruSeq v3 single-read flow cell and a 50-cycle single-read sequence run were performed on an Illumina HiSeq instrument. Reads were aligned to a sequence database containing the human genome (build GRCh37/hg19, February 2009, from genome.ucsc.edu) and alignment was performed using Novoalign v2.08.01. Total gene counts were calculated for each exon and untranslated region (UTR) of the genes using gene coordinates obtained from http://genome.ucsc.edu. Genes that were not expressed in our RNA-Seq data or for which the expression was missing for most of the samples were excluded from further analysis.^15^

### MicroRNA

The Agilent Human miRNA Microarray V19.0 (Agilent Technologies Inc., Santa Clara, CA, USA) was used. Data were required to pass QC parameters established by Agilent that included tests for excessive background fluorescence, excessive variation among probe sequence replicates on the array, and measures of the total gene signal on the array to assess low signal. Samples failing to meet quality standards were relabeled, hybridized to arrays, and rescanned. If a sample failed QC assessment a second time, the sample was excluded from analysis. The repeatability associated with this microarray was extremely high (*r* = .98)^12^; comparison of miRNA expression levels obtained from the Agilent microarray with those obtained from quantitative PCR had an agreement of 100% in terms of directionality of findings and the FCs were almost identical.^16^ To normalize differences in miRNA expression that could be attributed to the array, amount of RNA, location on array, or factors that could erroneously influence miRNA expression levels, total gene signal was normalized by multiplying each sample by a scaling factor which was the median of the 75th percentiles of all the samples divided by the individual 75th percentile of each sample.^17^

### MAPK-signaling genes

The KEGG (www.genome.jp/kegg/pathway/hsa/hsa04010.html) pathway map for MAPK signaling was used to identify genes. We identified 255 genes (Supplemental Table S1) in this signaling pathway; 241 of these genes had sufficient expression in CRC tissue for statistical analysis.

### Statistical methods

We performed negative binomial mixed effects modeling in SAS (accounting for carcinoma/normal status as well as subject effect) to determine which genes in the MAPK-signaling pathway had a statistically significant difference in expression between individually paired CRC and normal mucosa and their related FC. We offset the overall exposure as the log of the expression of all identified protein-coding genes in the negative binomial model (n = 17 461). The Benjamini and Hochberg^18^ method was used to control the false discovery rate (FDR) with a value of <0.05. An FC greater than 1 indicates a positive differential expression (ie, upregulated in carcinoma tissue); an FC between 0 and 1 indicates a negative differential expression (ie, downregulated in carcinoma tissue). We calculated the level of expression of each gene by dividing the total expression for that gene in an individual by the total expression of all protein-coding genes per million transcripts (RPMPCG or reads per million protein-coding genes). We considered overall CRC differential expression as well as differential expression for microsatellite unstable (MSI) and stable (MSS) tumors separately.

We focused on those genes and miRNAs with FCs of >1.50 or <0.67 to have find meaningful biological differences between carcinoma and normal samples. There were 814 miRNAs expressed in greater than 20% of normal colorectal mucosa samples that were analyzed; differential expression was calculated using subject-level paired data as the expression in the carcinoma tissue minus the expression in the normal mucosa. In these analyses, we fit a least squares linear regression model to the RPMPCG differential expression levels and miRNA differential expression levels. *P* values were generated using the bootstrap method by creating a distribution of 10 000 *F* statistics derived by resampling the residuals from the null hypothesis model of no association between gene expression and miRNA expression using the boot package in R. Linear models were adjusted for age and sex. Multiplicity adjustments for gene/miRNA associations were made at the gene level using the FDR by Benjamini and Hochberg.^18^

### Bioinformatics analysis

We analyzed significantly associated genes with FC of <0.67 and >1.50 for seed region matches with miRNAs. The mRNA 3ʹ UTR FASTA as well as the seed region sequence of the associated miRNA was analyzed to determine seed region pairings between miRNA and mRNA. MicroRNA seed regions were calculated as described in our previous work^19^; we included seeds of 6, 7, and 8 nucleotides in length. A seed match would increase the likelihood that an identified miRNA:mRNA interaction was more likely to have a direct biological effect on expression given a higher propensity for binding. Because miRTarBase^20^ uses findings from many different investigations and alignments, we used FASTA sequences generated from both GRCh37 and GRCh38 *Homo sapiens*, using UCSC Table Browser (https://genome.ucsc.edu/cgi-bin/hgTables).^21^ FASTA sequences that matched our Ensembl IDs and had consensus coding sequences available were downloaded. Scripts in R 3.2.3 and in perl 5.018002 were used to conduct the analysis.

## Results

The study population comprised 77.9% of participants who were diagnosed with first primary colon cancer and 22.1% with first primary rectal cancer (Table 1). Most of the study participants were men (54.4%) and non-Hispanic white (74.2%). The most common tumor mutation was in *TP53* (47.5%); 13.4% had an MSI tumor.

**Table 1.** Description of study population.

	No. (%)
Site	
Colon	169 (77.9)
Rectal	48 (22.1)
Sex	
Male	118 (54.4)
Female	99 (45.6)
Age	
Mean (SD)	64.8 (10.1)
Race	
Non-Hispanic white	161 (74.2)
Hispanic	14 (6.5)
Non-Hispanic black	8 (3.7)
Unknown	34 (15.7)
Tumor phenotype	
*TP53* mutated	103 (47.5)
*KRAS* mutated	69 (31.8)
*BRAF*-mutated	21 (10.1)
CIMP high	45 (20.7)
MSI	29 (13.4)

Abbreviations: CIMP, CpG island methylator phenotype; MSI, microsatellite unstable.

**Figure 1.**
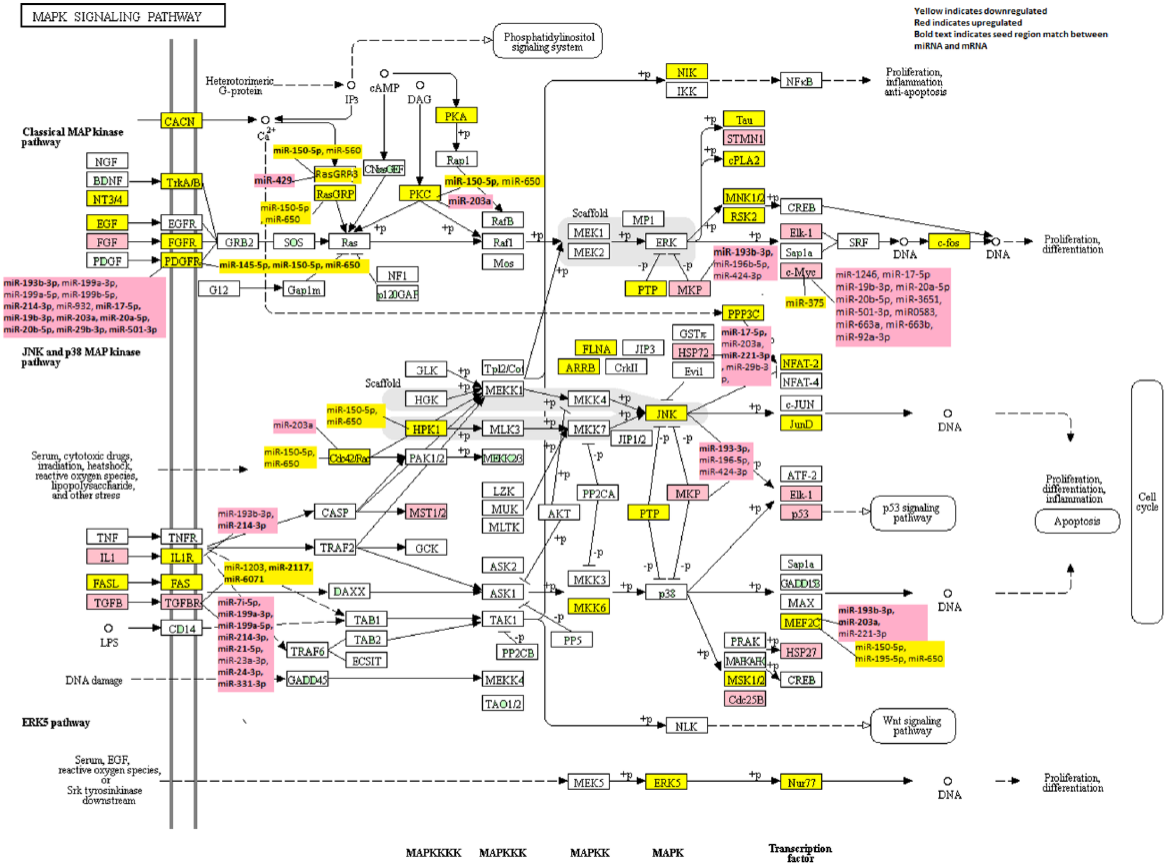
Dysregulated genes and their associated microRNAs in the KEGG MAPK-signaling pathway in colorectal cancer.

Of the 241 genes analyzed in the KEGG MAPK pathway, 83 (34.4%) were statistically significantly dysregulated in CRC overall with an FC of >1.50 or <0.67 (Table 2; Supplemental Table S2 has a list of all genes in the pathway). Of these 83 dysregulated genes, 60 were downregulated and 23 were upregulated. The most downregulated genes were fibroblast growth factor 9 (*FGF9*) (FC 0.18), protein phosphatase 3 regulatory subunit B (*PPP3R2*) (FC 0.21), and 2 calcium channel voltage-dependent genes subunit gamma 3 and 7 (*CACNG3* and *CACNG7*) (FC 0.25). Interestingly, 2 of the most upregulated genes were also FGFs (*FGF20* FC 5.29 and *FGF19* FC 9.42). The gene that was most upregulated in carcinomas was *CACNG4* (FC 3.30). Figure 1 illustrates the up- and downregulated genes in the MAPK pathway.

**Table 2.** Significant differentially expressed genes in the MAPK-signaling pathway where fold change is <0.67 or >1.50.

Gene name	Tumor mean	Normal mean	Fold change	*P* value	Adjusted *P* value
Downregulated					
* FGF9*	1.66	9.08	0.18	4.27E−30	6.44E−29
* PPP3R2*	0.20	0.93	0.21	3.56E−07	8.02E−07
* CACNG3*	0.16	0.62	0.25	4.46E−04	7.32E−04
* CACNG7*	0.17	0.67	0.25	1.91E−05	3.48E−05
* CACNA1A*	3.27	12.58	0.26	2.27E−32	4.57E−31
* CACNA1G*	1.26	4.80	0.26	4.08E−16	1.79E−15
* CACNA1F*	1.68	6.25	0.27	1.60E−21	1.01E−20
* RASGRP2*	6.72	24.73	0.27	1.04E−31	1.93E−30
* PRKCB*	14.65	53.12	0.28	1.52E−41	7.31E−40
* CACNA1I*	1.91	6.56	0.29	8.80E−19	4.51E−18
* MAPK10*	9.26	28.10	0.33	8.93E−34	1.96E−32
* MAP4K1*	7.24	21.46	0.34	1.58E−26	1.74E−25
* CACNG2*	0.21	0.57	0.37	2.28E−03	3.41E−03
* PRKACB*	62.60	162.73	0.38	5.72E−40	1.97E−38
* FGFR2*	26.51	68.82	0.39	2.32E−21	1.40E−20
* MEF2C*	27.15	66.82	0.41	1.00E−41	6.02E−40
* FOS*	185.52	453.52	0.41	9.65E−31	1.66E−29
* CACNG1*	0.27	0.65	0.42	3.35E−05	5.89E−05
* FGF10*	1.70	4.06	0.42	1.85E−10	5.45E−10
* PTPN5*	0.89	2.09	0.42	7.08E−07	1.55E−06
* DUSP1*	67.66	154.53	0.44	1.02E−34	2.46E−33
* EGF*	2.83	6.34	0.45	1.71E−05	3.15E−05
* CACNB2*	26.71	59.55	0.45	1.00E−38	3.03E−37
* IL1R2*	7.02	15.61	0.45	1.00E−13	3.77E−13
* NTRK1*	1.10	2.43	0.45	3.24E−07	7.36E−07
* CACNA1B*	1.79	3.91	0.46	1.99E−06	4.14E−06
* NTRK2*	17.81	38.41	0.46	1.04E−08	2.67E−08
* DUSP5*	28.27	59.40	0.48	1.39E−22	9.28E−22
* CACNA1H*	53.11	106.08	0.50	5.22E−27	5.99E−26
* FGFR3*	36.15	70.78	0.51	1.13E−18	5.54E−18
* MAPT*	4.51	8.74	0.52	1.01E−12	3.40E−12
* NFATC1*	12.01	22.97	0.52	3.23E−17	1.50E−16
* PDGFRA*	88.47	166.61	0.53	5.08E−26	5.11E−25
* CACNA2D3*	1.56	2.91	0.53	1.46E−04	2.46E−04
* MAPK7*	21.28	38.75	0.55	6.78E−29	9.08E−28
* FAS*	28.16	50.95	0.55	2.75E−24	2.46E−23
* CACNA1S*	0.56	1.00	0.56	6.90E−03	1.00E−02
* RASGRP1*	21.77	38.90	0.56	1.20E−22	8.23E−22
* PRKACG*	0.17	0.31	0.56	1.84E−03	2.79E−03
* RASGRP3*	25.53	45.22	0.56	1.16E−20	6.81E−20
* FGF2*	10.04	17.66	0.57	2.79E−13	9.89E−13
* FGF13*	2.42	4.18	0.58	2.84E−05	5.06E−05
* vRAC2*	22.11	37.39	0.59	4.70E−16	2.02E−15
* FGF7*	8.17	13.70	0.60	1.49E−08	3.69E−08
* NTF3*	0.84	1.41	0.60	1.04E−03	1.61E−03
* RPS6KA1*	89.25	148.07	0.60	1.24E−30	2.00E−29
* JUND*	127.78	210.71	0.61	1.88E−36	5.04E−35
* PLA2G4F*	22.98	37.48	0.61	2.67E−10	7.76E−10
* CACNA2D2*	10.30	16.63	0.62	1.02E−08	2.63E−08
* RPS6KA5*	18.57	29.87	0.62	1.84E−15	7.76E−15
* FLNC*	46.98	75.44	0.62	3.90E−09	1.02E−08
* ARRB1*	52.99	84.74	0.63	4.44E−24	3.61E−23
* NR4A1*	201.17	319.66	0.63	5.51E−11	1.72E−10
* FASLG*	1.07	1.70	0.63	4.96E−03	7.29E−03
* IL1R1*	57.71	89.40	0.65	9.55E−17	4.34E−16
* HSPA2*	7.29	11.21	0.65	5.51E−06	1.08E−05
* PTPN7*	16.22	24.78	0.65	3.08E−08	7.57E−08
* MAP3K14*	33.86	51.73	0.65	1.46E−24	1.35E−23
* MAP2K6*	25.29	38.55	0.66	1.60E−10	4.77E−10
* MKNK1*	63.58	95.95	0.66	2.04E−27	2.46E−26
Upregulated					
* HSPB1*	60.24	40.01	1.51	3.87E−12	1.24E−11
* TGFBR1*	106.44	70.13	1.52	6.14E−19	3.21E−18
* STK3*	41.26	26.65	1.55	1.64E−18	7.88E−18
* HSPA6*	5.49	3.43	1.60	3.67E−05	6.40E−05
* ELK1*	49.96	30.79	1.62	4.90E−24	3.81E−23
* DUSP10*	27.04	16.22	1.67	3.95E−14	1.59E−13
* FGF1*	4.77	2.79	1.71	9.30E−06	1.75E−05
* TP53*	105.07	59.63	1.76	3.25E−24	2.79E−23
* HSPA1B*	102.12	55.34	1.85	4.49E−24	3.61E−23
* PDGFRB*	166.24	87.76	1.89	8.03E−28	1.02E−26
* TGFB2*	9.23	4.52	2.04	8.04E−14	3.13E−13
* FGF18*	1.90	0.85	2.22	1.27E−05	2.36E−05
* HSPA8*	631.28	273.40	2.31	5.34E−56	1.29E−53
* CACNG8*	7.57	3.27	2.32	7.89E−10	2.19E−09
* STMN1*	77.81	31.07	2.50	1.15E−40	4.63E−39
* DUSP4*	56.69	21.44	2.64	2.53E−22	1.65E−21
* CDC25B*	169.88	60.96	2.79	9.71E−54	7.80E−52
* IL1A*	7.56	2.39	3.17	2.02E−19	1.08E−18
* NTF4*	0.55	0.17	3.23	3.80E−06	7.57E−06
* CACNG4*	3.41	1.03	3.30	8.11E−10	2.22E−09
* MYC*	181.11	49.00	3.70	5.76E−55	6.94E−53
* FGF20*	0.72	0.14	5.29	2.29E−05	4.15E−05
* FGF19*	1.39	0.15	9.42	6.08E−13	2.12E−12

Because more than 85% of tumors were considered MSS, it is not surprising that findings for mRNA expression in CRCs and for MSS-specific cancers were almost identical (see Supplemental Table S3 for MSS-specific associations). Five genes, *CACNB4* (FC_MSS_ 0.65), *MAP3K6* (FC_MSS_ 0.66), *CD14* (FC_MSS_ 0.66), *CACNA2D4* (FC_MSS_ 0.66), and *MAP4K4* (FC_MSS_ 1.52) had slightly stronger FC for MSS-specific tumors than for all tumor combined (FC_overall_ 0.67, 0.73, 0.68, 0.67, and 1.47, respectively). More differences between MSI-specific tumors and all tumors were observed as might be expected (see Supplemental Table S4 for MSI-specific associations). Although *CACNB4, MAP3K6, CD14*, and *CACNA2D4* were not associated with differential expression in MSI tumors, 7 genes were significantly downregulated for MSI-specific tumors only (*RPS6KA6* FC 0.20, *PLA2G4E* FC 0.30, *RASGFR1* FC 0.39, *PLA2G4C* FC 0.47, *MAPK8IP1* FC 0.47, *PDGFA* FC 0.53, and *TGFBR2* FC 0.61). Four other genes were significantly upregulated only for MSI-specific tumors (*DUSP7* FC 1.57, *HRAS* FC 1.92, *IL1B* FC 2.06, and *PLA2G4A* FC 2.70). However, 13 genes associated with significant higher expression in carcinoma compared with normal mucosa were not significantly upregulated in MSI tumors (*HSPB1, TGFBR1, ELK1, FGF1, PDGFRB, TGFBR2, FDF18, CACNG8, NTF4, CACNG4, FGF20, ILA1A*, and *FGF19*).

Of the 99 dysregulated genes identified overall or for MSS- or MSI-specific tumors, 13 were associated with miRNAs for a total of 68 miRNA:mRNA associations after adjustment for multiple comparisons (Table 3). Several genes were associated with 2 or 3 miRNAs, such as *RASGRP2, MAP4K1, IL1R1, DUSP4, RAC2, RASGRP3*, and *PRKCB. HSPA8* was associated with 5 miRNAs, whereas *PDGFRB* and *MEF2C* were associated with 6 miRNAs. *TGFBR1* (11 miRNAs), *PDGFRA* (10 miRNAs), and *MYC* (12 miRNAs) had the most associations with miRNAs. Thirteen of the miRNAs:mRNA seed region matches (*MEF2C* with miR-203a; *TGFBR1* with miR-2117 and miR-6071; *DUSP4* with miR-193b-3p; *PDGFRA* with miR-17-5p, miR-19b-3p, miR-203a, miR-20a-5p, miR-20b-5p, miR-29b-3p, miR-501-3p; *RASGRP3* with miR-429; and *PRKCB* and miR-203a) were inversely associated suggesting a greater likelihood of a direct effect between the miRNA and mRNA. Notably, none of the miRNAs associated with *MYC* had a seed region match, whereas all of the miRNAs associated with *PDGFRA* had a seed region match. Many miRNAs were associated with multiple mRNAs (Table 4). Both miR-150-5p and miR-650 were associated with the same 7 genes. Three of these genes had a seed region match for miR-150-5p (*PDGFRA, RASGRP3*, and *PRKCB*), whereas miR-650 only had a seed region match with *PDGFRA*. MiR-203a was associated with 5 mRNAs, 3 of which (*MEF2C, PDGFRA*, and *PRKCB*) had a seed region match. MiR-193b-3p was associated with 4 genes, 3 of which had a seed region match (*MEF2C, PDGFRB*, and *DUSP4*). Figure 1 also shows these miRNA:mRNA associations in the KEGG MAPK-signaling pathway.

**Table 3.** Associations between MAPK pathway–dysregulated mRNA and miRNAs.

Gene name	Tumor mean	Normal mean	Fold change	MiRNA	Tumor mean	Normal mean	Fold change	β	Raw *P* value	FDR *P* value
*RASGRP2*	6.72	24.73	0.27	hsa-miR-150-5p	14.90	39.17	0.38	.31	<.0001	.0407
				hsa-miR-650	4.51	16.60	0.27	.29	.0002	.0407
*MEF2C*	27.15	66.82	0.41	hsa-miR-150-5p	14.90	39.17	0.38	.35	<.0001	.0116
				**hsa-miR-193b-3p**	9.12	5.42	1.68	.28	<.0001	.0116
				hsa-miR-195-5p	3.59	12.18	0.29	.25	.0004	.025
				**hsa-miR-203a**	12.52	3.70	3.38	−.30	<.0001	.0116
				hsa-miR-221-3p	13.53	4.12	3.28	−.26	.0002	.0148
				hsa-miR-650	4.51	16.60	0.27	.34	<.0001	.0116
*MAP4K1*	7.24	21.46	0.34	hsa-miR-150-5p	14.90	39.17	0.38	.30	<.0001	.0203
				hsa-miR-650	4.51	16.60	0.27	.30	.0003	.0488
*TGFBR1*	106.44	70.13	1.52	**hsa-let-7i-5p**	62.16	39.97	1.56	.25	.0006	.031
				hsa-miR-1203	1.76	2.83	0.62	−.23	.0007	.031
				**hsa-miR-199a-3p**	44.83	22.53	1.99	.24	.0007	.031
				**hsa-miR-199a-5p**	20.18	9.28	2.17	.22	.0016	.0458
				**hsa-miR-2117**	1.50	4.09	0.37	−.22	.0011	.0358
				**hsa-miR-214-3p**	13.24	6.13	2.16	.26	.0005	.031
				**hsa-miR-21-5p**	463.11	167.37	2.77	.25	.0006	.031
				hsa-miR-23a-3p	174.68	87.53	2.00	.25	.0008	.031
				**hsa-miR-24-3p**	106.75	62.39	1.71	.24	.0009	.0318
				**hsa-miR-331-3p**	14.64	9.30	1.57	.24	.0006	.031
				**hsa-miR-6071**	0.97	1.70	0.57	−.22	.0017	.0458
*HSPA8*	631.28	273.40	2.31	**hsa-miR-17-5p**	61.04	16.38	3.73	.27	.0003	.0407
				hsa-miR-203a	12.52	3.70	3.38	.35	<.0001	.0271
				**hsa-miR-221-3p**	13.53	4.12	3.28	.28	<.0001	.0271
				hsa-miR-29b-3p	24.31	9.83	2.47	.27	.0003	.0407
				**hsa-miR-93-5p**	41.72	15.20	2.74	.25	.0003	.0407
*PDGFRB*	166.24	87.76	1.89	**hsa-miR-193b-3p**	9.12	5.42	1.68	.27	.0002	.0181
				hsa-miR-199a-3p	44.83	22.53	1.99	.33	<.0001	.0102
				hsa-miR-199a-5p	20.18	9.28	2.17	.35	<.0001	.0102
				hsa-miR-199b-5p	4.69	1.53	3.07	.34	<.0001	.0102
				**hsa-miR-214-3p**	13.24	6.13	2.16	.38	<.0001	.0102
				hsa-miR-934	4.36	0.94	4.66	.46	<.0001	.0102
*IL1R1*	57.71	89.40	0.65	hsa-miR-193b-3p	9.12	5.42	1.68	.31	<.0001	.0203
				**hsa-miR-214-3p**	13.24	6.13	2.16	.27	.0003	.0407
*DUSP4*	56.69	21.44	2.64	**hsa-miR-193b-3p**	9.12	5.42	1.68	−.27	<.0001	.0163
				hsa-miR-196b-5p	17.89	5.53	3.24	−.31	<.0001	.0163
				hsa-miR-424-3p	39.81	25.37	1.57	−.29	.0003	.0271
*RAC2*	22.11	37.39	0.59	hsa-miR-150-5p	14.90	39.17	0.38	.39	<.0001	.0203
				hsa-miR-203a	12.52	3.70	3.38	−.28	<.0001	.0203
				hsa-miR-650	4.51	16.60	0.27	.36	<.0001	.0203
*PDGFRA*	88.47	166.61	0.53	**hsa-miR-145-5p**	132.97	223.14	0.60	.26	.0002	.0163
				**hsa-miR-150-5p**	14.90	39.17	0.38	.24	.0004	.0233
				**hsa-miR-17-5p**	61.04	16.38	3.73	−.28	<.0001	.0136
				**hsa-miR-19b-3p**	29.80	10.42	2.86	−.29	<.0001	.0136
				**hsa-miR-203a**	12.52	3.70	3.38	−.30	<.0001	.0136
				**hsa-miR-20a-5p**	70.78	17.61	4.02	−.28	.0002	.0163
				**hsa-miR-20b-5p**	17.65	3.30	5.35	−.30	<.0001	.0136
				**hsa-miR-29b-3p**	24.31	9.83	2.47	−.27	.0003	.0222
				**hsa-miR-501-3p**	7.07	2.95	2.39	−.25	.0004	.0233
				**hsa-miR-650**	4.51	16.60	0.27	.32	<.0001	.0136
*MYC*	181.11	49.00	3.70	hsa-miR-1246	629.21	412.81	1.52	.27	.0002	.0163
				hsa-miR-17-5p	61.04	16.38	3.73	.35	<.0001	.0136
				hsa-miR-19b-3p	29.80	10.42	2.86	.27	.0002	.0163
				hsa-miR-20a-5p	70.78	17.61	4.02	.33	<.0001	.0136
				hsa-miR-20b-5p	17.65	3.30	5.35	.31	.0002	.0163
				hsa-miR-3651	58.66	25.92	2.26	.28	.0003	.0188
				hsa-miR-375	20.50	54.53	0.38	−.29	<.0001	.0136
				hsa-miR-501-3p	7.07	2.95	2.39	.26	.0003	.0188
				hsa-miR-583	6.61	3.22	2.05	.26	.0004	.0233
				hsa-miR-663a	374.83	234.91	1.60	.28	.0003	.0188
				hsa-miR-663b	65.50	32.21	2.03	.33	<.0001	.0136
				hsa-miR-92a-3p	121.60	41.18	2.95	.32	<.0001	.0136
*RASGRP3*	25.53	45.22	0.56	**hsa-miR-150-5p**	14.90	39.17	0.38	.35	<.0001	.0203
				**hsa-miR-429**	13.33	8.29	1.61	−.25	.0003	.0407
				hsa-miR-650	4.51	16.60	0.27	.35	<.0001	.0203
*PRKCB*	14.65	53.12	0.28	**hsa-miR-150-5p**	14.90	39.17	0.38	.38	<.0001	.0102
				**hsa-miR-203a**	12.52	3.70	3.38	−.30	<.0001	.0102
				hsa-miR-650	4.51	16.60	0.27	.35	<.0001	.0102

Abbreviations: FDR, false discovery rate; mRNA, messenger RNA; miRNA, microRNA.

Bold text indicates seed region match between miRNA and mRNA.

**Table 4.** MiRNAs in MAPK pathway associated with mRNA by seed region match.

MiRNA	With seed region match	Without seed region match
hsa-let-7i-5p	*TGFBR1*	
hsa-miR-1203		*TGFBR1*
hsa-miR-1246		*MYC*
hsa-miR-145-5p	*PDGFRA*	
hsa-miR-150-5p (7)	*PDGFRA, RASGRP3, PRKCB*	*RASGRP2, MEF2C, MAP4K1, RAC2*,
hsa-miR-17-5p (3)	*HSPA8*, ***PDGFRA***	*MYC*
hsa-miR-193b-3p (4)	***MEF2C***, *PDGFRB, DUSP4*	*IL1R*
hsa-miR-195-5p		*MEF2C*
hsa-miR-196b-5p		*DUSP4*
hsa-miR-199a-3p (2)	*TGFBR1*	*PDGFRB*
has-miR-199a-5p	*TGFBR1*	*PDGFRB*
hsa-miR-199b-5p		*PDGFRB*
hsa-miR-19b-3p (2)	***PDGFRA***	*MYC*
hsa-miR-203a (5)	***MEF2C***, ***PDGFRA***, ***PRKCB***	*HSPA8, RAC2*
hsa-miR-20a-5p (2)	***PDGFRA***	*MYC*
hsa-miR-20b-5p (2)	***PDGFRA***	*MYC*
hsa-miR-2117	***TGFBR1***	
hsa-miR-214-3p (3)	*TGFBR1, PDGFRB, IL1R1*	
hsa-miR-21-5p	*TGFBR1*	
hsa-miR-221-3p (2)	*HSPA8*	*MEF2C*
hsa-miR-23a-3p		*TGFBR1*
hsa-miR-24-3p	*TGFBR1*	
hsa-miR-29b-3p (2)	***PDGFRA***	*HSPA8*,
hsa-miR-331-3p	*TGFBR1*	
hsa-miR-3651		*MYC*
hsa-miR-375		*MYC*
hsa-miR-424-3p		*DUSP4*
hsa-miR-429	***RASGRP3***	
hsa-miR-501-3p (2)	***PDGFRA***	*MYC*
hsa-miR-583		*MYC*
hsa-miR-6071	***TGFBR1***	
hsa-miR-650 (7)	*PDGFRA*	*RASGRP1, MEF2C, MAP4K1, RAC2, RASGRP3, PRKCB*
hsa-miR-663a		*MYC*
hsa-miR-663b		*MYC*
hsa-miR-92a-3p		*MYC*
hsa-miR-934		*PDGFRB*
hsa-miR-93-5p	*HSPA8*	

Abbreviations: mRNA, messenger RNA; miRNA, microRNA.

Bold text indicates negative β coefficient between miRNA and mRNA for those with a seed region match; numbers in parentheses indicate number of associations identified.

## Discussion

Roughly 41% of genes in the MAPK-signaling pathway were dysregulated in CRCs when considering all cancers as well as MSI and MSS tumors. Genes both upstream and downstream of *ERK1*/*ERK2, JNK, p38*, and *ERK5* were dysregulated, implying that all MAPK-signaling pathway arms may be involved in CRC. Of the 99 dysregulated genes, 13 were associated with miRNAs and 13 of the 68 miRNA:mRNA associations had both a seed region match with a negative β coefficient between the differential expression of the miRNA and mRNA, suggesting a greater likelihood that these genes were directly influenced by miRNA binding. We focus our discussion mainly on those genes and their associated miRNAs given their greater probability of being useful targets for examining prognosis or therapeutic agents.

Given the number of associations observed between miRNAs and mRNAs in the MAPK-signaling pathway, interpretation of results can be difficult. Seed region matches between the miRNA and the 3ʹUTR of the mRNA can increase the likelihood that binding occurs that can alter the gene expression. However, other associations between miRNAs and mRNAs in the MAPK-signaling pathway, such as those in which there is a positive β coefficient or no identified seed match, suggest an indirect biological effect. Indirect effects most likely operate through a feedback or feed forward loop.^22–24^ In feedback loops, regulators such as miRNAs can have either the same effect (repression of expression) or opposite effects on each other.^23^ In feed forward loops, a TF regulates the miRNA and the target gene (TG), which in turn is regulated by the miRNA. In this instance, the miRNA may regulate the TG directly, through seed region binding leading to mRNA degradation or translational repression, or indirectly, through repression of the TF that is influencing transcription of the same TG. Studies suggest that regulatory pathways involving miRNAs are prevalent mechanisms of altering gene expression.^23^

Examination of the MAPK-signaling pathway focusing on those miRNA:mRNA associations that have a greater likelihood of a direct effect, given both a seed region match and an inverse association between the differentially expressed miRNA and mRNA, may help provide focus on targets that may be the most rewarding in terms of future research relating to targeted therapeutics. Within each of the major components of the signaling pathway, ie, ERK1/2, JNK, and p38, we identified miRNA:mRNA associations of potential importance. In the classical arm of the pathway (see Figure 1), several factors including the growth hormone *PDGFRA, RASGRP3* which can alter immune function as well as activate RAS genes, and *PRKCB*, a protein kinase involved in numerous cell functions including apoptosis and cell proliferation, had associations with miRNAs that suggested a direct biological effect. *PDGFRA* was inversely associated with 7 miRNAs, 5 of which were in the miR17-92 cluster as well as with miR-203a and miR-501-3p. Differential expression of *PRKCB* was inversely associated with differential expression of miR-203a. Downstream of these associations, several of these miRNAs were associated with *DUSP4* and *MYC*, suggesting an indirect effect between miRNAs and these genes. Upregulation of miR-203, as in our CRC data, has been shown to inhibit proliferation and metastasis in CRC.^25^ Studies support that miR-150 and the miR17-92 cluster are functionally involved in immune function and cell survival and proliferation.^26^

Others have reviewed components of the *ERK1*/*ERK2* MAPK pathway that are associated with various cancers.^27^ Several miRNAs were identified as targeting *EGFR* and RAS genes. Of these miRNAs, miR-145 previously was associated with *EGFR*, and we identified this miRNA as also being associated with *PDGFRA*. As our analysis of miRNA:mRNA interactions focused only on those genes that were significantly differently expressed at levels >1.50 or <0.67, we did not assess miRNAs with *EGFR* (FC 0.81) or any of the RAS genes specifically. Our findings instead focused on miRNA:mRNA associations that could alter RAS gene function because of downstream effects. Our findings along with those of Masliah-Planchon et al^27^ suggest the need for further research into miRNA:mRNA interactions that appear to have a direct effect and may yield useful biomarkers that can be used for prognosis as well as therapeutic purposes. Previous studies have shown that higher *TRBP* expression increased expression of miR-17 and miR-20a, both which were upregulated in our data^4^; *TRBP* is regulated by the MAPK/ERK pathway^4^ suggesting another indirect effect between genes in this signaling pathway and miRNAs.

The JNK/p38 arm of the KEGG MAPK-signaling pathway also had miRNA:mRNA associations that would imply a direct effect. Some associations, including *IL1R1* with miR-214-3p and *TFGBR1* with miR-2217 and miR-6071, were upstream of *JNK* and *p38*, implying an impact on factors that alter activation of the pathway. Other direct biological associations were observed between *MEF2C* and miR-193b-3p and miR-203a, downstream of *p38*. Again these direct biological associations suggest potential targets for future research. *IL1R* has previously been associated with miR-378,^27^ although we did not see an association with this miRNA in our CRC data. Other studies have shown that miR-125a-3p can upregulate p38 in orofacial tissue^28^; however, we also did not see an association between this miRNA and any genes within the p38 arm of the pathway.

Our study represents one of the largest reported to date that includes paired expression levels for both miRNAs and mRNA; however, the sample size is still considered small to examine several factors including their effects on prognosis. A strength is our paired samples of carcinoma and normal mucosa, although it should be recognized that normal mucosa may not be truly “normal.” Despite this limitation, the normal mucosa samples we used were the closest normal mucosa that could be obtained for a matched paired analysis. In addition, normal colonic mucosa was taken from the same colonic site as the tumor and the miRNA and mRNA data were generated from the same RNA prep. We did not examine all statistically significant genes that were differentially expressed and miRNAs, but focused on those that also had an FC of >1.50 or <0.67. Using these cut points to define further analysis with miRNAs, we could have missed genes associated with miRNAs. We exclusively used the KEGG pathway database to identify signaling pathway genes, and genes not identified in KEGG that may be related to the pathway and have an impact of expression of both miRNA and mRNA were not examined. An important limitation in our data in evaluating the impact of miRNA on genes in the pathway was our use of mRNA expression data. Because miRNAs could have part or even most of their impact posttranscriptionally, we could have missed important associations. However, much of the current information on miRNA TGs comes from gene expression data. At any rate, observed associations may still have important biological meaning, but it must be acknowledged that important associations could have been missed.^20,29^

In conclusion, we show that the MAPK-signaling pathway is dysregulated in CRC and that several dysregulated genes in this pathway are associated with miRNAs. Some inverse associations were noted with negative β coefficients and seed region matches between the miRNA and mRNA, suggesting that these miRNAs directly repress gene expression and thus alter MAPK signaling. Other associations, that were both downstream or upstream of these direct biological miRNA:mRNA associations, may further result in disruption of the pathway. We encourage others to both replicate these findings and validate results in targeted laboratory experiments that can help solidify important therapeutic targets.

## Supplemental Material

Supplemental_Tables – Supplemental material for The MAPK-Signaling Pathway in Colorectal Cancer: Dysregulated Genes and Their Association With MicroRNAs Click here for additional data file. Supplemental material, Supplemental_Tables for The MAPK-Signaling Pathway in Colorectal Cancer: Dysregulated Genes and Their Association With MicroRNAs by Martha L Slattery, Lila E Mullany, Lori C Sakoda, Roger K Wolff, Wade S Samowitz and Jennifer S Herrick in Cancer Informatics

## References

[1] Imajo M Tsuchiya Y Nishida E. Regulatory mechanisms and functions of MAP kinase signaling pathways. IUBMB Life. 2006;58:312–317. 16754324 10.1080/15216540600746393

[2] Marshall MS. Ras target proteins in eukaryotic cells. FASEB J. 1995;9:1311–1318. 7557021 10.1096/fasebj.9.13.7557021

[3] Qi M Elion EA. MAP kinase pathways. J Cell Sci. 2005;118:3569–3572. 16105880 10.1242/jcs.02470

[4] Paroo Z Ye X Chen S Liu Q. Phosphorylation of the human microRNA-generating complex mediates MAPK/Erk signaling. Cell. 2009;139:112–122. 19804757 10.1016/j.cell.2009.06.044 PMC2760040

[5] Heo I Kim VN. Regulating the regulators: posttranslational modifications of RNA silencing factors. Cell. 2009;139:28–31. 19804751 10.1016/j.cell.2009.09.013

[6] Zhu QY Liu Q Chen JX Lan K Ge BX. MicroRNA-101 targets MAPK phosphatase-1 to regulate the activation of MAPKs in macrophages. J Immunol. 2010;185:7435–7442. 21068409 10.4049/jimmunol.1000798

[7] Schmitt DC Madeira da Silva L Zhang W et al ErbB2-intronic microRNA-4728: a novel tumor suppressor and antagonist of oncogenic MAPK signaling. Cell Death Dis. 2015;6:e1742. 25950472 10.1038/cddis.2015.116 PMC4669696

[8] Mutlu M Saatci O Ansari SA et al miR-564 acts as a dual inhibitor of PI3K and MAPK signaling networks and inhibits proliferation and invasion in breast cancer. Sci Rep. 2016;6:32541. 27600857 10.1038/srep32541 PMC5013276

[9] Burotto M Chiou VL Lee JM Kohn EC. The MAPK pathway across different malignancies: a new perspective. Cancer. 2014;120:3446–3456. 24948110 10.1002/cncr.28864 PMC4221543

[10] Slattery ML Potter J Caan B et al Energy balance and colon cancer—beyond physical activity. Cancer Res. 1997;57:75–80. 8988044

[11] Slattery ML Caan BJ Benson J Murtaugh M. Energy balance and rectal cancer: an evaluation of energy intake, energy expenditure, and body mass index. Nutr Cancer. 2003;46:166–171. 14690792 10.1207/S15327914NC4602_09

[12] Slattery ML Herrick JS Pellatt DF et al MicroRNA profiles in colorectal carcinomas, adenomas and normal colonic mucosa: variations in miRNA expression and disease progression. Carcinogenesis. 2016;37:245–261. 26740022 10.1093/carcin/bgv249 PMC4766359

[13] Slattery ML Herrick JS Mullany LE et al An evaluation and replication of miRNAs with disease stage and colorectal cancer-specific mortality. Int J Cancer. 2015;137:428–438. 25484364 10.1002/ijc.29384 PMC4428989

[14] Pellatt AJ Slattery ML Mullany LE Wolff RK Pellatt DF. Dietary intake alters gene expression in colon tissue: possible underlying mechanism for the influence of diet on disease. Pharmacogenet Genomics. 2016;26:294–306. 26959716 10.1097/FPC.0000000000000217 PMC4853256

[15] Slattery ML Pellatt DF Mullany LE Wolff RK Herrick JS. Gene expression in colon cancer: A focus on tumor site and molecular phenotype. Gene Chromosome Canc. 2015;54:527–541. 10.1002/gcc.22265 PMC5998821 26171582

[16] Pellatt DF Stevens JR Wolff RK et al Expression profiles of miRNA subsets distinguish human colorectal carcinoma and normal colonic mucosa. Clin Transl Gastroenterol. 2016;7:e152. 26963002 10.1038/ctg.2016.11 PMC4822091

[17] Agilent Technologies Inc. Agilent GeneSpring User Manual. Santa Clara, CA: Agilent Technologies Inc; 2013.

[18] Benjamini Y Hochberg Y. Controlling the false discovery rate: a practical and powerful approach to multiple testing. J R Stat Soc. 1995;57:289–300.

[19] Mullany LE Herrick JS Wolff RK Slattery ML. MicroRNA seed region length impact on target messenger RNA expression and survival in colorectal cancer. PLoS ONE. 2016;11:e0154177. 27123865 10.1371/journal.pone.0154177 PMC4849741

[20] Chou CH Chang NW Shrestha S et al miRTarBase 2016: updates to the experimentally validated miRNA-target interactions database. Nucleic Acids Res. 2016;44:D239–147. 26590260 10.1093/nar/gkv1258 PMC4702890

[21] Karolchik D Hinrichs AS Furey TS et al The UCSC Table Browser data retrieval tool. Nucleic Acids Res. 2004;32:D493–D496. 14681465 10.1093/nar/gkh103 PMC308837

[22] Lin Y Zhang Q Zhang HM et al Transcription factor and miRNA co-regulatory network reveals shared and specific regulators in the development of B cell and T cell. Sci Rep. 2015;5:15215. 26487345 10.1038/srep15215 PMC4613730

[23] Martinez NJ Walhout AJ. The interplay between transcription factors and microRNAs in genome-scale regulatory networks. Bioessays. 2009;31:435–445. 19274664 10.1002/bies.200800212 PMC3118512

[24] Mangan S Alon U. Structure and function of the feed-forward loop network motif. Proc Natl Acad Sci U S A. 2003;100:11980–11985. 14530388 10.1073/pnas.2133841100 PMC218699

[25] Gao B Yu T Xue D et al A multidimensional integration analysis reveals potential bridging targets in the process of colorectal cancer liver metastasis. PLoS ONE. 2017;12:e0178760. 28628609 10.1371/journal.pone.0178760 PMC5476238

[26] Hoefig KP Heissmeyer V. MicroRNAs grow up in the immune system. Curr Opin Immunol. 2008;20:281–287. 18554884 10.1016/j.coi.2008.05.005

[27] Masliah-Planchon J Garinet S Pasmant E. RAS-MAPK pathway epigenetic activation in cancer: miRNAs in action. Oncotarget. 2016;7:38892–38907. 26646588 10.18632/oncotarget.6476 PMC5122439

[28] Dong Y Li P Ni Y Zhao J Liu Z. Decreased microRNA-125a-3p contributes to upregulation of p38 MAPK in rat trigeminal ganglions with orofacial inflammatory pain. PLoS ONE. 2014;9:e111594. 25380251 10.1371/journal.pone.0111594 PMC4224409

[29] Slattery ML Herrick JS Stevens JR Wolff RK Mullany LE. An assessment of database-validated microRNA target genes in normal colonic mucosa: implications for pathway analysis. Cancer Inform. 2017;16. doi: 10.1177/1176935117716405. PMC5484592 28690395

