# Supplementary material for: The MAPK-Signaling Pathway in Colorectal Cancer: Dysregulated Genes and Their Association With MicroRNAs
Source: Cancer Inform. 2018 Mar 26;17:1176935118766522. doi: 10.1177/1176935118766522 (PMC5888819; doi:10.1177/1176935118766522)
Supplement: Supplemental_Tables – Supplemental material for The MAPK-Signaling Pathway in Colorectal Cancer: Dysregulated Genes and Their Association With MicroRNAs [file Supplemental_Tables.pdf]

Supplemental Table S1. Genes identified and analyzed in KEGG MAPK Signaling Pathway

Gene ID	Gene Name	Description
773	*CACNA1A*	calcium voltage-gated channel subunit alpha1 A [KO:K04344]
774	*CACNA1B*	calcium voltage-gated channel subunit alpha1 B [KO:K04849]
775	*CACNA1C*	calcium voltage-gated channel subunit alpha1 C [KO:K04850]
776	*CACNA1D*	calcium voltage-gated channel subunit alpha1 D [KO:K04851]
777	*CACNA1E*	calcium voltage-gated channel subunit alpha1 E [KO:K04852]
778	*CACNA1F*	calcium voltage-gated channel subunit alpha1 F [KO:K04853]
8913	*CACNA1G*	calcium voltage-gated channel subunit alpha1 G [KO:K04854]
8912	*CACNA1H*	calcium voltage-gated channel subunit alpha1 H [KO:K04855]
8911	*CACNA1I*	calcium voltage-gated channel subunit alpha1 I [KO:K04856]
779	*CACNA1S*	calcium voltage-gated channel subunit alpha1 S [KO:K04857]
781	*CACNA2D1*	calcium voltage-gated channel auxiliary subunit alpha2delta 1 [KO:K04858]
9254	*CACNA2D2*	calcium voltage-gated channel auxiliary subunit alpha2delta 2 [KO:K04859]
55799	*CACNA2D3*	calcium voltage-gated channel auxiliary subunit alpha2delta 3 [KO:K04860]
93589	*CACNA2D4*	calcium voltage-gated channel auxiliary subunit alpha2delta 4 [KO:K04861]
782	*CACNB1*	calcium voltage-gated channel auxiliary subunit beta 1 [KO:K04862]
783	*CACNB2*	calcium voltage-gated channel auxiliary subunit beta 2 [KO:K04863]
784	*CACNB3*	calcium voltage-gated channel auxiliary subunit beta 3 [KO:K04864]
785	*CACNB4*	calcium voltage-gated channel auxiliary subunit beta 4 [KO:K04865]
786	*CACNG1*	calcium voltage-gated channel auxiliary subunit gamma 1 [KO:K04866]
10369	*CACNG2*	calcium voltage-gated channel auxiliary subunit gamma 2 [KO:K04867]
10368	*CACNG3*	calcium voltage-gated channel auxiliary subunit gamma 3 [KO:K04868]
27092	*CACNG4*	calcium voltage-gated channel auxiliary subunit gamma 4 [KO:K04869]
27091	*CACNG5*	calcium voltage-gated channel auxiliary subunit gamma 5 [KO:K04870]
59285	*CACNG6*	calcium voltage-gated channel auxiliary subunit gamma 6 [KO:K04871]
59284	*CACNG7*	calcium voltage-gated channel auxiliary subunit gamma 7 [KO:K04872]
59283	*CACNG8*	calcium voltage-gated channel auxiliary subunit gamma 8 [KO:K04873]
5566	*PRKACA*	protein kinase cAMP-activated catalytic subunit alpha [KO:K04345] [EC:2.7.11.11]
5567	*PRKACB*	protein kinase cAMP-activated catalytic subunit beta [KO:K04345] [EC:2.7.11.11]
5568	*PRKACG*	protein kinase cAMP-activated catalytic subunit gamma [KO:K04345] [EC:2.7.11.11]
5578	*PRKCA*	protein kinase C alpha [KO:K02677] [EC:2.7.11.13]
5579	*PRKCB*	protein kinase C beta [KO:K19662] [EC:2.7.11.13]
5582	*PRKCG*	protein kinase C gamma [KO:K19663] [EC:2.7.11.13]
2768	*GNA12*	G protein subunit alpha 12 [KO:K04346]
55970	*GNG12*	G protein subunit gamma 12 [KO:K04347]
5530	*PPP3CA*	protein phosphatase 3 catalytic subunit alpha [KO:K04348] [EC:3.1.3.16]
5532	*PPP3CB*	protein phosphatase 3 catalytic subunit beta [KO:K04348] [EC:3.1.3.16]
5533	*PPP3CC*	protein phosphatase 3 catalytic subunit gamma [KO:K04348] [EC:3.1.3.16]
5534	*PPP3R1*	protein phosphatase 3 regulatory subunit B, alpha [KO:K06268]
5535	*PPP3R2*	protein phosphatase 3 regulatory subunit B, beta [KO:K06268]
5923	*RASGRF1*	Ras protein specific guanine nucleotide releasing factor 1 [KO:K04349]
5924	*RASGRF2*	Ras protein specific guanine nucleotide releasing factor 2 [KO:K12326]
10125	*RASGRP1*	RAS guanyl releasing protein 1 [KO:K04350]
10235	*RASGRP2*	RAS guanyl releasing protein 2 [KO:K12361]
25780	*RASGRP3*	RAS guanyl releasing protein 3 [KO:K12362]
115727	*RASGRP4*	RAS guanyl releasing protein 4 [KO:K12363]
9693	*RAPGEF2*	Rap guanine nucleotide exchange factor 2 [KO:K08018]
4763	*NF1*	neurofibromin 1 [KO:K08052]
5921	*RASA1*	RAS p21 protein activator 1 [KO:K04352]
5922	*RASA2*	RAS p21 protein activator 2 [KO:K08053]
5906	*RAP1A*	RAP1A, member of RAS oncogene family [KO:K04353]
5908	*RAP1B*	RAP1B, member of RAS oncogene family [KO:K07836]

4803	*NGF*	nerve growth factor [KO:K02582]
627	*BDNF*	brain derived neurotrophic factor [KO:K04355]
4908	*NTF3*	neurotrophin 3 [KO:K04356]
4909	*NTF4*	neurotrophin 4 [KO:K12457]
1950	*EGF*	epidermal growth factor [KO:K04357]
2246	*FGF1*	fibroblast growth factor 1 [KO:K18496]
2247	*FGF2*	fibroblast growth factor 2 [KO:K18497]
2248	*FGF3*	fibroblast growth factor 3 [KO:K04358]
2249	*FGF4*	fibroblast growth factor 4 [KO:K04358]
8822	*FGF17*	fibroblast growth factor 17 [KO:K04358]
2251	*FGF6*	fibroblast growth factor 6 [KO:K04358]
2252	*FGF7*	fibroblast growth factor 7 [KO:K04358]
2253	*FGF8*	fibroblast growth factor 8 [KO:K04358]
2254	*FGF9*	fibroblast growth factor 9 [KO:K04358]
2255	*FGF10*	fibroblast growth factor 10 [KO:K04358]
2256	*FGF11*	fibroblast growth factor 11 [KO:K04358]
2257	*FGF12*	fibroblast growth factor 12 [KO:K04358]
2258	*FGF13*	fibroblast growth factor 13 [KO:K04358]
2259	*FGF14*	fibroblast growth factor 14 [KO:K04358]
8823	*FGF16*	fibroblast growth factor 16 [KO:K04358]
2250	*FGF5*	fibroblast growth factor 5 [KO:K04358]
8817	*FGF18*	fibroblast growth factor 18 [KO:K04358]
9965	*FGF19*	fibroblast growth factor 19 [KO:K04358]
26281	*FGF20*	fibroblast growth factor 20 [KO:K04358]
26291	*FGF21*	fibroblast growth factor 21 [KO:K04358]
27006	*FGF22*	fibroblast growth factor 22 [KO:K04358]
8074	*FGF23*	fibroblast growth factor 23 [KO:K04358]
5154	*PDGFA*	platelet derived growth factor subunit A [KO:K04359]
5155	*PDGFB*	platelet derived growth factor subunit B [KO:K17386]
4914	*NTRK1*	neurotrophic receptor tyrosine kinase 1 [KO:K03176] [EC:2.7.10.1]
4915	*NTRK2*	neurotrophic receptor tyrosine kinase 2 [KO:K04360] [EC:2.7.10.1]
1956	*EGFR*	epidermal growth factor receptor [KO:K04361] [EC:2.7.10.1]
2260	*FGFR1*	fibroblast growth factor receptor 1 [KO:K04362] [EC:2.7.10.1]
2263	*FGFR2*	fibroblast growth factor receptor 2 [KO:K05093] [EC:2.7.10.1]
2261	*FGFR3*	fibroblast growth factor receptor 3 [KO:K05094] [EC:2.7.10.1]
2264	*FGFR4*	fibroblast growth factor receptor 4 [KO:K05095] [EC:2.7.10.1]
5156	*PDGFRA*	platelet derived growth factor receptor alpha [KO:K04363] [EC:2.7.10.1]
5159	*PDGFRB*	platelet derived growth factor receptor beta [KO:K05089] [EC:2.7.10.1]
2885	*GRB2*	growth factor receptor bound protein 2 [KO:K04364]
6654	*SOS1*	SOS Ras/Rac guanine nucleotide exchange factor 1 [KO:K03099]
6655	*SOS2*	SOS Ras/Rho guanine nucleotide exchange factor 2 [KO:K03099]
3265	*HRAS*	HRas proto-oncogene, GTPase [KO:K02833]
3845	*KRAS*	KRAS proto-oncogene, GTPase [KO:K07827]
4893	*NRAS*	NRAS proto-oncogene, GTPase [KO:K07828]
6237	*RRAS*	related RAS viral (r-ras) oncogene homolog [KO:K07829]
22800	*RRAS2*	related RAS viral (r-ras) oncogene homolog 2 [KO:K07830]
22808	*MRAS*	muscle RAS oncogene homolog [KO:K07831]
673	*BRAF*	B-Raf proto-oncogene, serine/threonine kinase [KO:K04365] [EC:2.7.11.1]
5894	*RAF1*	Raf-1 proto-oncogene, serine/threonine kinase [KO:K04366] [EC:2.7.11.1]
4342	*MOS*	MOS proto-oncogene, serine/threonine kinase [KO:K04367] [EC:2.7.11.1]
5604	*MAP2K1*	mitogen-activated protein kinase kinase 1 [KO:K04368] [EC:2.7.12.2]
5605	*MAP2K2*	mitogen-activated protein kinase kinase 2 [KO:K04369] [EC:2.7.12.2]
8649	*LAMTOR3*	late endosomal/lysosomal adaptor, MAPK and MTOR activator 3 [KO:K04370]

5594	*MAPK1*	mitogen-activated protein kinase 1 [KO:K04371] [EC:2.7.11.24]
5595	*MAPK3*	mitogen-activated protein kinase 3 [KO:K04371] [EC:2.7.11.24]
8569	*MKNK1*	MAP kinase interacting serine/threonine kinase 1 [KO:K04372] [EC:2.7.11.1]
2872	*MKNK2*	MAP kinase interacting serine/threonine kinase 2 [KO:K04372] [EC:2.7.11.1]
6197	*RPS6KA3*	ribosomal protein S6 kinase A3 [KO:K04373] [EC:2.7.11.1]
6195	*RPS6KA1*	ribosomal protein S6 kinase A1 [KO:K04373] [EC:2.7.11.1]
6196	*RPS6KA2*	ribosomal protein S6 kinase A2 [KO:K04373] [EC:2.7.11.1]
27330	*RPS6KA6*	ribosomal protein S6 kinase A6 [KO:K04373] [EC:2.7.11.1]
468	*ATF4*	activating transcription factor 4 [KO:K04374]
2002	*ELK1*	ELK1, ETS transcription factor [KO:K04375]
2005	*ELK4*	ELK4, ETS transcription factor [KO:K04376]
4609	*MYC*	MYC proto-oncogene, bHLH transcription factor [KO:K04377]
6722	*SRF*	serum response factor [KO:K04378]
2353	*FOS*	Fos proto-oncogene, AP-1 transcription factor subunit [KO:K04379]
4137	*MAPT*	microtubule associated protein tau [KO:K04380]
3925	*STMN1*	stathmin 1 [KO:K04381]
123745	*PLA2G4E*	phospholipase A2 group IVE [KO:K16342] [EC:3.1.1.4]
5321	*PLA2G4A*	phospholipase A2 group IVA [KO:K16342] [EC:3.1.1.4]
8681	*JMJD7-PLA2G4B*	JMJD7-PLA2G4B readthrough [KO:K16342] [EC:3.1.1.4]
100137049	*PLA2G4B*	phospholipase A2 group IVB [KO:K16342] [EC:3.1.1.4]
8605	*PLA2G4C*	phospholipase A2 group IVC [KO:K16342] [EC:3.1.1.4]
283748	*PLA2G4D*	phospholipase A2 group IVD [KO:K16342] [EC:3.1.1.4]
255189	*PLA2G4F*	phospholipase A2 group IVF [KO:K16342] [EC:3.1.1.4]
7124	*TNF*	tumor necrosis factor [KO:K03156]
3552	*IL1A*	interleukin 1 alpha [KO:K04383]
3553	*IL1B*	interleukin 1 beta [KO:K04519]
7040	*TGFB1*	transforming growth factor beta 1 [KO:K13375]
7042	*TGFB2*	transforming growth factor beta 2 [KO:K13376]
7043	*TGFB3*	transforming growth factor beta 3 [KO:K13377]
7132	*TNFRSF1A*	TNF receptor superfamily member 1A [KO:K03158]
3554	*IL1R1*	interleukin 1 receptor type 1 [KO:K04386]
7850	*IL1R2*	interleukin 1 receptor type 2 [KO:K04387]
7046	*TGFBR1*	transforming growth factor beta receptor 1 [KO:K04674] [EC:2.7.11.30]
7048	*TGFBR2*	transforming growth factor beta receptor 2 [KO:K04388] [EC:2.7.11.30]
356	*FASLG*	Fas ligand [KO:K04389]
355	*FAS*	Fas cell surface death receptor [KO:K04390]
929	*CD14*	CD14 molecule [KO:K04391]
5879	*RAC1*	ras-related C3 botulinum toxin substrate 1 (rho family, small GTP binding protein Rac1) [KO:K04392]
5880	*RAC2*	ras-related C3 botulinum toxin substrate 2 (rho family, small GTP binding protein Rac2) [KO:K07860]
5881	*RAC3*	ras-related C3 botulinum toxin substrate 3 (rho family, small GTP binding protein Rac3) [KO:K07861]
998	*CDC42*	cell division cycle 42 [KO:K04393]
836	*CASP3*	caspase 3 [KO:K02187] [EC:3.4.22.56]
7186	*TRAF2*	TNF receptor associated factor 2 [KO:K03173] [EC:2.3.2.27]
1616	*DAXX*	death domain associated protein [KO:K02308]
7189	*TRAF6*	TNF receptor associated factor 6 [KO:K03175] [EC:2.3.2.27]
1647	*GADD45A*	growth arrest and DNA damage inducible alpha [KO:K04402]
4616	*GADD45B*	growth arrest and DNA damage inducible beta [KO:K04402]
10912	*GADD45G*	growth arrest and DNA damage inducible gamma [KO:K04402]
10454	*TAB1*	TGF-beta activated kinase 1 (MAP3K7) binding protein 1 [KO:K04403]
23118	*TAB2*	TGF-beta activated kinase 1/MAP3K7 binding protein 2 [KO:K04404]

51295	*ECSIT*	ECSIT signalling integrator [KO:K04405]
8491	*MAP4K3*	mitogen-activated protein kinase kinase kinase kinase 3 [KO:K04406] [EC:2.7.11.1]
9448	*MAP4K4*	mitogen-activated protein kinase kinase kinase kinase 4 [KO:K04407] [EC:2.7.11.1]
11184	*MAP4K1*	mitogen-activated protein kinase kinase kinase kinase 1 [KO:K04408] [EC:2.7.11.1]
5058	*PAK1*	p21 (RAC1) activated kinase 1 [KO:K04409] [EC:2.7.11.1]
5062	*PAK2*	p21 (RAC1) activated kinase 2 [KO:K04410] [EC:2.7.11.1]
6789	*STK4*	serine/threonine kinase 4 [KO:K04411]
6788	*STK3*	serine/threonine kinase 3 [KO:K04412]
5871	*MAP4K2*	mitogen-activated protein kinase kinase kinase kinase 2 [KO:K04414] [EC:2.7.11.1]
1326	*MAP3K8*	mitogen-activated protein kinase kinase kinase 8 [KO:K04415] [EC:2.7.11.25]
4214	*MAP3K1*	mitogen-activated protein kinase kinase kinase 1 [KO:K04416] [EC:2.7.11.25]
4296	*MAP3K11*	mitogen-activated protein kinase kinase kinase 11 [KO:K04419] [EC:2.7.11.25]
10746	*MAP3K2*	mitogen-activated protein kinase kinase kinase 2 [KO:K04420] [EC:2.7.11.25]
4215	*MAP3K3*	mitogen-activated protein kinase kinase kinase 3 [KO:K04421] [EC:2.7.11.25]
9175	*MAP3K13*	mitogen-activated protein kinase kinase kinase 13 [KO:K04422] [EC:2.7.11.25]
7786	*MAP3K12*	mitogen-activated protein kinase kinase kinase 12 [KO:K04423] [EC:2.7.11.25]
51776	*MAP3K20*	mitogen-activated protein kinase kinase kinase 20 [KO:K04424] [EC:2.7.11.25]
9064	*MAP3K6*	mitogen-activated protein kinase kinase kinase 6 [KO:K04425] [EC:2.7.11.25]
4217	*MAP3K5*	mitogen-activated protein kinase kinase kinase 5 [KO:K04426] [EC:2.7.11.25]
6885	*MAP3K7*	mitogen-activated protein kinase kinase kinase 7 [KO:K04427] [EC:2.7.11.25]
4216	*MAP3K4*	mitogen-activated protein kinase kinase kinase 4 [KO:K04428] [EC:2.7.11.25]
9344	*TAOK2*	TAO kinase 2 [KO:K04429] [EC:2.7.11.1]
51347	*TAOK3*	TAO kinase 3 [KO:K04429] [EC:2.7.11.1]
57551	*TAOK1*	TAO kinase 1 [KO:K04429] [EC:2.7.11.1]
6416	*MAP2K4*	mitogen-activated protein kinase kinase 4 [KO:K04430] [EC:2.7.12.2]
5609	*MAP2K7*	mitogen-activated protein kinase kinase 7 [KO:K04431] [EC:2.7.12.2]
5606	*MAP2K3*	mitogen-activated protein kinase kinase 3 [KO:K04432] [EC:2.7.12.2]
5608	*MAP2K6*	mitogen-activated protein kinase kinase 6 [KO:K04433] [EC:2.7.12.2]
9479	*MAPK8IP1*	mitogen-activated protein kinase 8 interacting protein 1 [KO:K04434]
23542	*MAPK8IP2*	mitogen-activated protein kinase 8 interacting protein 2 [KO:K04435]
23162	*MAPK8IP3*	mitogen-activated protein kinase 8 interacting protein 3 [KO:K04436]
2316	*FLNA*	filamin A [KO:K04437]
2318	*FLNC*	filamin C [KO:K04437]
2317	*FLNB*	filamin B [KO:K04437]
1398	*CRK*	CRK proto-oncogene, adaptor protein [KO:K04438]
1399	*CRKL*	CRK like proto-oncogene, adaptor protein [KO:K04438]
408	*ARRB1*	arrestin beta 1 [KO:K04439]
409	*ARRB2*	arrestin beta 2 [KO:K04439]
5599	*MAPK8*	mitogen-activated protein kinase 8 [KO:K04440] [EC:2.7.11.24]
5602	*MAPK10*	mitogen-activated protein kinase 10 [KO:K04440] [EC:2.7.11.24]
5601	*MAPK9*	mitogen-activated protein kinase 9 [KO:K04440] [EC:2.7.11.24]
5600	*MAPK11*	mitogen-activated protein kinase 11 [KO:K04441] [EC:2.7.11.24]
6300	*MAPK12*	mitogen-activated protein kinase 12 [KO:K04441] [EC:2.7.11.24]
5603	*MAPK13*	mitogen-activated protein kinase 13 [KO:K04441] [EC:2.7.11.24]
1432	*MAPK14*	mitogen-activated protein kinase 14 [KO:K04441] [EC:2.7.11.24]
8550	*MAPKAPK5*	mitogen-activated protein kinase-activated protein kinase 5 [KO:K04442] [EC:2.7.11.1]
9261	*MAPKAPK2*	mitogen-activated protein kinase-activated protein kinase 2 [KO:K04443] [EC:2.7.11.1]
7867	*MAPKAPK3*	mitogen-activated protein kinase-activated protein kinase 3 [KO:K04444] [EC:2.7.11.1]
9252	*RPS6KA5*	ribosomal protein S6 kinase A5 [KO:K04445] [EC:2.7.11.1]
8986	*RPS6KA4*	ribosomal protein S6 kinase A4 [KO:K16510] [EC:2.7.11.1]
994	*CDC25B*	cell division cycle 25B [KO:K05866] [EC:3.1.3.48]
4772	*NFATC1*	nuclear factor of activated T-cells 1 [KO:K04446]
4775	*NFATC3*	nuclear factor of activated T-cells 3 [KO:K17333]

3725	*JUN*	Jun proto-oncogene, AP-1 transcription factor subunit [KO:K04448]
3727	*JUND*	JunD proto-oncogene, AP-1 transcription factor subunit [KO:K04449]
1386	*ATF2*	activating transcription factor 2 [KO:K04450]
7157	*TP53*	tumor protein p53 [KO:K04451]
1649	*DDIT3*	DNA damage inducible transcript 3 [KO:K04452]
4149	*MAX*	MYC associated factor X [KO:K04453]
4208	*MEF2C*	myocyte enhancer factor 2C [KO:K04454]
3315	*HSPB1*	heat shock protein family B (small) member 1 [KO:K04455]
207	*AKT1*	AKT serine/threonine kinase 1 [KO:K04456] [EC:2.7.11.1]
208	*AKT2*	AKT serine/threonine kinase 2 [KO:K04456] [EC:2.7.11.1]
10000	*AKT3*	AKT serine/threonine kinase 3 [KO:K04456] [EC:2.7.11.1]
5494	*PPM1A*	protein phosphatase, Mg2+/Mn2+ dependent 1A [KO:K04457] [EC:3.1.3.16]
5801	*PTPRR*	protein tyrosine phosphatase, receptor type R [KO:K04458] [EC:3.1.3.48]
84867	*PTPN5*	protein tyrosine phosphatase, non-receptor type 5 [KO:K18018] [EC:3.1.3.48]
5778	*PTPN7*	protein tyrosine phosphatase, non-receptor type 7 [KO:K18019] [EC:3.1.3.48]
1843	*DUSP1*	dual specificity phosphatase 1 [KO:K21278] [EC:3.1.3.48 3.1.3.16]
1846	*DUSP4*	dual specificity phosphatase 4 [KO:K04459] [EC:3.1.3.48 3.1.3.16]
1844	*DUSP2*	dual specificity phosphatase 2 [KO:K04459] [EC:3.1.3.48 3.1.3.16]
1849	*DUSP7*	dual specificity phosphatase 7 [KO:K04459] [EC:3.1.3.48 3.1.3.16]
1850	*DUSP8*	dual specificity phosphatase 8 [KO:K04459] [EC:3.1.3.48 3.1.3.16]
1847	*DUSP5*	dual specificity phosphatase 5 [KO:K04459] [EC:3.1.3.48 3.1.3.16]
80824	*DUSP16*	dual specificity phosphatase 16 [KO:K04459] [EC:3.1.3.48 3.1.3.16]
1848	*DUSP6*	dual specificity phosphatase 6 [KO:K21946] [EC:3.1.3.48 3.1.3.16]
1852	*DUSP9*	dual specificity phosphatase 9 [KO:K18498] [EC:3.1.3.48 3.1.3.16]
11221	*DUSP10*	dual specificity phosphatase 10 [KO:K20216] [EC:3.1.3.48 3.1.3.16]
1845	*DUSP3*	dual specificity phosphatase 3 [KO:K17614] [EC:3.1.3.48 3.1.3.16]
5536	*PPP5C*	protein phosphatase 5 catalytic subunit [KO:K04460] [EC:3.1.3.16]
100506012	*PPP5D1*	PPP5 tetratricopeptide repeat domain containing 1 [KO:K04460] [EC:3.1.3.16]
5495	*PPM1B*	protein phosphatase, Mg2+/Mn2+ dependent 1B [KO:K04461] [EC:3.1.3.16]
3312	*HSPA8*	heat shock protein family A (Hsp70) member 8 [KO:K03283]
3303	*HSPA1A*	heat shock protein family A (Hsp70) member 1A [KO:K03283]
3306	*HSPA2*	heat shock protein family A (Hsp70) member 2 [KO:K03283]
3305	*HSPA1L*	heat shock protein family A (Hsp70) member 1 like [KO:K03283]
3304	*HSPA1B*	heat shock protein family A (Hsp70) member 1B [KO:K03283]
3310	*HSPA6*	heat shock protein family A (Hsp70) member 6 [KO:K03283]
2122	*MECOM*	MDS1 and EVI1 complex locus [KO:K04462]
5607	*MAP2K5*	mitogen-activated protein kinase kinase 5 [KO:K04463] [EC:2.7.12.2]
5598	*MAPK7*	mitogen-activated protein kinase 7 [KO:K04464] [EC:2.7.11.24]
3164	*NR4A1*	nuclear receptor subfamily 4 group A member 1 [KO:K04465]
9020	*MAP3K14*	mitogen-activated protein kinase kinase kinase 14 [KO:K04466] [EC:2.7.11.25]
1147	*CHUK*	conserved helix-loop-helix ubiquitous kinase [KO:K04467] [EC:2.7.11.10]
3551	*IKBKB*	inhibitor of nuclear factor kappa B kinase subunit beta [KO:K07209] [EC:2.7.11.10]
8517	*IKBKG*	inhibitor of nuclear factor kappa B kinase subunit gamma [KO:K07210]
51701	*NLK*	nemo like kinase [KO:K04468] [EC:2.7.11.24]
4790	*NFKB1*	nuclear factor kappa B subunit 1 [KO:K02580]
4791	*NFKB2*	nuclear factor kappa B subunit 2 [KO:K04469]
5970	*RELA*	RELA proto-oncogene, NF-kB subunit [KO:K04735]
5971	*RELB*	RELB proto-oncogene, NF-kB subunit [KO:K09253]

Supplemental Table S2. Differentially expression of genes in MAPK-Signaling Pathway and CRC

Gene Name	Tumor Mean	Normal Mean	Fold Change	P-Value	Adjusted P-Value
FGF9	1.66	9.08	0.18	4.27E-30	6.44E-29
PPP3R2	0.20	0.93	0.21	3.56E-07	8.02E-07
CACNG3	0.16	0.62	0.25	4.46E-04	7.32E-04
CACNG7	0.17	0.67	0.25	1.91E-05	3.48E-05
CACNA1A	3.27	12.58	0.26	2.27E-32	4.57E-31
CACNA1G	1.26	4.80	0.26	4.08E-16	1.79E-15
CACNA1F	1.68	6.25	0.27	1.60E-21	1.01E-20
RASGRP2	6.72	24.73	0.27	1.04E-31	1.93E-30
PRKCB	14.65	53.12	0.28	1.52E-41	7.31E-40
CACNA1I	1.91	6.56	0.29	8.80E-19	4.51E-18
MAPK10	9.26	28.10	0.33	8.93E-34	1.96E-32
MAP4K1	7.24	21.46	0.34	1.58E-26	1.74E-25
CACNG2	0.21	0.57	0.37	2.28E-03	3.41E-03
PRKACB	62.60	162.73	0.38	5.72E-40	1.97E-38
FGFR2	26.51	68.82	0.39	2.32E-21	1.40E-20
MEF2C	27.15	66.82	0.41	1.00E-41	6.02E-40
FOS	185.52	453.52	0.41	9.65E-31	1.66E-29
CACNG1	0.27	0.65	0.42	3.35E-05	5.89E-05
FGF10	1.70	4.06	0.42	1.85E-10	5.45E-10
PTPN5	0.89	2.09	0.42	7.08E-07	1.55E-06
DUSP1	67.66	154.53	0.44	1.02E-34	2.46E-33
EGF	2.83	6.34	0.45	1.71E-05	3.15E-05
CACNB2	26.71	59.55	0.45	1.00E-38	3.03E-37
IL1R2	7.02	15.61	0.45	1.00E-13	3.77E-13
NTRK1	1.10	2.43	0.45	3.24E-07	7.36E-07
CACNA1B	1.79	3.91	0.46	1.99E-06	4.14E-06
NTRK2	17.81	38.41	0.46	1.04E-08	2.67E-08
DUSP5	28.27	59.40	0.48	1.39E-22	9.28E-22
CACNA1H	53.11	106.08	0.50	5.22E-27	5.99E-26
FGFR3	36.15	70.78	0.51	1.13E-18	5.54E-18
MAPT	4.51	8.74	0.52	1.01E-12	3.40E-12
NFATC1	12.01	22.97	0.52	3.23E-17	1.50E-16
PDGFRA	88.47	166.61	0.53	5.08E-26	5.11E-25
CACNA2D3	1.56	2.91	0.53	1.46E-04	2.46E-04
MAPK7	21.28	38.75	0.55	6.78E-29	9.08E-28
FAS	28.16	50.95	0.55	2.75E-24	2.46E-23
CACNA1S	0.56	1.00	0.56	6.90E-03	1.00E-02
RASGRP1	21.77	38.90	0.56	1.20E-22	8.23E-22
PRKACG	0.17	0.31	0.56	1.84E-03	2.79E-03
RASGRP3	25.53	45.22	0.56	1.16E-20	6.81E-20
FGF2	10.04	17.66	0.57	2.79E-13	9.89E-13
FGF13	2.42	4.18	0.58	2.84E-05	5.06E-05
RAC2	22.11	37.39	0.59	4.70E-16	2.02E-15
FGF7	8.17	13.70	0.60	1.49E-08	3.69E-08
NTF3	0.84	1.41	0.60	1.04E-03	1.61E-03
RPS6KA1	89.25	148.07	0.60	1.24E-30	2.00E-29
JUND	127.78	210.71	0.61	1.88E-36	5.04E-35
PLA2G4F	22.98	37.48	0.61	2.67E-10	7.76E-10
CACNA2D2	10.30	16.63	0.62	1.02E-08	2.63E-08
RPS6KA5	18.57	29.87	0.62	1.84E-15	7.76E-15

FLNC	46.98	75.44	0.62	3.90E-09	1.02E-08
ARRB1	52.99	84.74	0.63	4.44E-24	3.61E-23
NR4A1	201.17	319.66	0.63	5.51E-11	1.72E-10
FASLG	1.07	1.70	0.63	4.96E-03	7.29E-03
IL1R1	57.71	89.40	0.65	9.55E-17	4.34E-16
HSPA2	7.29	11.21	0.65	5.51E-06	1.08E-05
PTPN7	16.22	24.78	0.65	3.08E-08	7.57E-08
MAP3K14	33.86	51.73	0.65	1.46E-24	1.35E-23
MAP2K6	25.29	38.55	0.66	1.60E-10	4.77E-10
MKNK1	63.58	95.95	0.66	2.04E-27	2.46E-26
CACNA2D4	8.65	12.94	0.67	1.05E-07	2.52E-07
BDNF	4.72	7.03	0.67	3.29E-06	6.60E-06
MAPK3	62.34	92.73	0.67	9.12E-19	4.58E-18
CACNB4	5.58	8.29	0.67	9.67E-05	1.64E-04
RASGRP4	5.22	7.74	0.67	9.38E-07	1.98E-06
CD14	9.64	14.13	0.68	1.36E-07	3.15E-07
FGFR1	54.37	77.12	0.70	6.72E-11	2.05E-10
RPS6KA6	10.53	14.89	0.71	9.47E-04	1.48E-03
RAP1B	72.62	101.88	0.71	1.01E-23	7.59E-23
SOS2	91.40	127.77	0.72	2.38E-20	1.33E-19
PDGFA	40.23	56.15	0.72	3.25E-11	1.03E-10
PLA2G4E	1.22	1.68	0.72	3.37E-02	4.51E-02
MAP3K6	21.20	29.17	0.73	9.95E-08	2.40E-07
FGF12	1.01	1.32	0.76	1.36E-01	1.70E-01
RAP1A	56.39	73.96	0.76	1.12E-13	4.16E-13
TNFRSF1A	90.65	118.74	0.76	4.32E-20	2.37E-19
MAP3K11	100.41	131.31	0.76	3.80E-16	1.69E-15
MECOM	154.07	196.91	0.78	1.58E-09	4.28E-09
MKNK2	184.21	235.23	0.78	8.60E-14	3.29E-13
CACNA2D1	12.45	15.82	0.79	1.52E-03	2.31E-03
AKT3	41.45	52.56	0.79	2.51E-05	4.51E-05
MAP2K5	29.19	36.52	0.80	4.08E-07	9.10E-07
RPS6KA2	43.83	54.74	0.80	3.04E-06	6.15E-06
PPM1A	116.19	144.84	0.80	2.47E-13	8.90E-13
DUSP8	41.88	52.20	0.80	7.25E-07	1.57E-06
EGFR	175.46	217.61	0.81	2.03E-13	7.42E-13
PLA2G4C	5.61	6.94	0.81	2.51E-02	3.42E-02
PPP3CC	17.08	20.97	0.81	4.27E-04	7.07E-04
FLNB	782.75	954.16	0.82	3.36E-10	9.63E-10
MAP3K12	48.60	59.03	0.82	1.14E-08	2.86E-08
FGF23	0.45	0.55	0.82	4.40E-01	4.91E-01
PRKCA	69.40	83.99	0.83	8.91E-08	2.17E-07
MAP3K13	84.75	101.91	0.83	2.01E-09	5.37E-09
MAP3K3	40.17	48.21	0.83	2.64E-06	5.40E-06
PPM1B	79.30	94.30	0.84	1.06E-08	2.69E-08
TAOK3	108.78	127.43	0.85	1.32E-07	3.10E-07
CRK	96.42	112.18	0.86	1.08E-07	2.56E-07
RELB	24.80	28.79	0.86	1.24E-03	1.91E-03
TAOK2	80.96	93.16	0.87	7.45E-07	1.60E-06
RRAS2	22.54	25.84	0.87	1.72E-02	2.38E-02
GADD45G	2.79	3.18	0.88	2.07E-01	2.50E-01
TAB2	128.75	146.44	0.88	8.45E-07	1.80E-06
CACNB1	14.72	16.72	0.88	4.31E-02	5.64E-02

PRKACA	53.45	60.68	0.88	3.93E-06	7.77E-06
TAB1	29.01	32.68	0.89	1.36E-03	2.09E-03
RASGRF1	2.50	2.81	0.89	4.14E-01	4.64E-01
RAF1	131.53	147.29	0.89	5.78E-07	1.28E-06
MAPK8IP3	129.73	144.99	0.89	2.01E-04	3.36E-04
GNG12	144.35	161.26	0.90	1.93E-03	2.91E-03
CACNA1E	6.12	6.79	0.90	3.93E-01	4.49E-01
PLA2G4B	38.29	42.23	0.91	1.99E-02	2.72E-02
MAX	70.54	77.59	0.91	2.51E-03	3.73E-03
MAP3K1	73.96	81.10	0.91	3.54E-03	5.23E-03
KRAS	107.64	117.88	0.91	1.65E-02	2.30E-02
MAP2K4	32.10	35.00	0.92	7.33E-02	9.30E-02
NFKB2	45.18	49.11	0.92	5.89E-02	7.59E-02
HSPA1L	2.74	2.97	0.92	3.64E-01	4.18E-01
MAP2K2	69.97	75.76	0.92	1.47E-02	2.08E-02
NFKB1	77.63	83.98	0.92	7.65E-03	1.10E-02
MAP3K5	68.22	73.59	0.93	3.82E-02	5.04E-02
JMJD7-PLA2G4B	66.55	71.72	0.93	3.74E-02	4.96E-02
IKBKB	137.36	145.80	0.94	6.63E-02	8.45E-02
TNF	1.95	2.04	0.96	7.52E-01	7.88E-01
PPP3CB	55.64	58.21	0.96	2.28E-01	2.74E-01
CASP3	42.94	44.90	0.96	3.39E-01	3.96E-01
PTPRR	11.77	12.30	0.96	7.01E-01	7.46E-01
MAP2K7	49.83	52.02	0.96	1.68E-01	2.06E-01
FGF11	3.97	4.12	0.96	7.29E-01	7.68E-01
PLA2G4A	6.99	7.23	0.97	8.04E-01	8.32E-01
SOS1	159.37	164.61	0.97	2.24E-01	2.70E-01
RASA1	127.03	130.76	0.97	2.90E-01	3.46E-01
ECSIT	18.73	19.16	0.98	6.83E-01	7.31E-01
FGF17	0.93	0.94	0.99	9.30E-01	9.30E-01
MAP2K3	71.67	72.13	0.99	8.80E-01	8.94E-01
MAPKAPK2	124.26	125.03	0.99	8.12E-01	8.35E-01
BRAF	55.05	55.31	1.00	8.86E-01	8.94E-01
MAP3K2	197.26	196.59	1.00	8.85E-01	8.94E-01
CACNB3	31.74	31.59	1.00	9.27E-01	9.30E-01
NFATC3	159.90	158.77	1.01	7.71E-01	8.05E-01
AKT1	153.83	152.34	1.01	7.08E-01	7.48E-01
LAMTOR3	30.88	30.52	1.01	8.00E-01	8.31E-01
CDC42	145.07	143.06	1.01	6.08E-01	6.60E-01
NLK	48.11	47.34	1.02	6.50E-01	6.99E-01
JUN	214.48	211.03	1.02	7.02E-01	7.46E-01
MAPK8	62.01	60.98	1.02	6.19E-01	6.69E-01
RASA2	81.22	79.82	1.02	5.99E-01	6.57E-01
MAPK11	6.71	6.59	1.02	8.23E-01	8.40E-01
PPP3CA	81.01	79.40	1.02	6.04E-01	6.59E-01
MAPK1	183.50	179.79	1.02	3.53E-01	4.09E-01
GRB2	114.66	112.16	1.02	4.07E-01	4.61E-01
MAPK9	65.92	64.12	1.03	4.12E-01	4.64E-01
DUSP6	81.99	79.35	1.03	4.78E-01	5.31E-01
TRAF6	38.06	36.78	1.03	3.37E-01	3.96E-01
MAPK12	46.73	44.85	1.04	3.25E-01	3.84E-01
PLA2G4D	1.06	1.01	1.05	8.14E-01	8.35E-01
TGFB1	41.60	39.64	1.05	3.65E-01	4.18E-01

FLNA	1070.76	1020.00	1.05	2.93E-01	3.48E-01
PPP5C	61.00	57.30	1.06	4.95E-02	6.45E-02
MAPK13	64.53	60.31	1.07	6.44E-02	8.25E-02
GADD45B	12.90	12.05	1.07	3.43E-01	4.00E-01
ARRB2	39.77	37.09	1.07	1.43E-01	1.78E-01
MAPK8IP1	7.51	6.98	1.08	4.07E-01	4.61E-01
MAP3K4	69.84	64.78	1.08	1.61E-02	2.25E-02
MAP3K8	22.11	20.49	1.08	1.84E-01	2.25E-01
ATF2	69.51	64.16	1.08	1.05E-02	1.49E-02
RAPGEF2	156.93	143.11	1.10	6.68E-04	1.06E-03
TGFBR2	197.09	178.93	1.10	5.25E-03	7.67E-03
FGF5	1.02	0.92	1.11	5.99E-01	6.57E-01
MAPK14	141.75	127.24	1.11	8.85E-05	1.51E-04
IKBKG	6.91	6.20	1.11	2.04E-01	2.49E-01
DUSP3	93.00	83.37	1.12	6.89E-04	1.09E-03
RELA	82.20	73.32	1.12	5.56E-06	1.08E-05
SRF	68.66	61.18	1.12	4.64E-04	7.46E-04
ELK4	257.18	228.38	1.13	1.63E-05	3.02E-05
RPS6KA3	249.75	221.67	1.13	5.35E-04	8.54E-04
PAK1	115.45	102.10	1.13	4.79E-05	8.31E-05
HSPA1A	29.65	26.18	1.13	1.23E-01	1.55E-01
MAP4K2	18.67	16.46	1.13	5.29E-02	6.85E-02
GADD45A	13.07	11.37	1.15	3.07E-02	4.13E-02
AKT2	156.81	135.87	1.15	2.66E-07	6.11E-07
DUSP2	8.36	7.24	1.15	1.11E-01	1.39E-01
FGF14	0.56	0.48	1.15	5.48E-01	6.06E-01
RRAS	19.63	16.97	1.16	7.41E-03	1.07E-02
MAP2K1	32.75	28.06	1.17	4.28E-04	7.07E-04
MAP3K7	72.49	62.09	1.17	9.69E-07	2.03E-06
TGFB3	16.83	14.40	1.17	3.72E-02	4.95E-02
CHUK	44.48	37.81	1.18	4.50E-04	7.32E-04
DUSP16	108.13	91.66	1.18	5.91E-06	1.14E-05
MAPK8IP2	2.34	1.95	1.20	1.54E-01	1.91E-01
IL1B	22.58	18.76	1.20	2.65E-02	3.58E-02
CACNA1C	70.88	58.66	1.21	4.64E-04	7.46E-04
ATF4	130.84	105.67	1.24	8.92E-13	3.03E-12
CRKL	130.15	103.92	1.25	3.08E-15	1.28E-14
DAXX	35.66	28.09	1.27	3.63E-10	1.03E-09
MAPKAPK5	60.52	47.19	1.28	1.05E-10	3.15E-10
CACNA1D	91.77	71.00	1.29	2.49E-06	5.13E-06
NF1	372.04	287.40	1.29	8.42E-25	8.12E-24
TAOK1	158.33	121.96	1.30	1.88E-20	1.08E-19
STK4	198.94	152.89	1.30	2.64E-12	8.70E-12
RPS6KA4	45.93	35.30	1.30	6.56E-11	2.03E-10
PDGFB	17.40	13.37	1.30	7.15E-05	1.23E-04
RASGRF2	31.70	24.17	1.31	8.81E-06	1.67E-05
DDIT3	11.07	8.29	1.34	3.01E-05	5.33E-05
RAC3	2.78	2.05	1.36	9.11E-03	1.30E-02
NRAS	104.64	76.74	1.36	3.26E-15	1.33E-14
TRAF2	40.20	29.46	1.36	8.02E-13	2.76E-12
FGFR4	68.96	50.37	1.37	2.80E-09	7.42E-09
DUSP7	23.89	17.23	1.39	5.23E-10	1.47E-09
MRAS	14.39	10.28	1.40	6.98E-06	1.34E-05

PAK2	186.93	132.40	1.41	6.02E-30	8.54E-29
RAC1	180.81	127.60	1.42	3.51E-26	3.67E-25
MAPKAPK3	44.98	31.68	1.42	5.84E-14	2.31E-13
MAP4K3	75.09	52.16	1.44	2.98E-17	1.41E-16
PPP3R1	89.41	61.65	1.45	1.55E-23	1.13E-22
GNA12	72.97	50.22	1.45	2.04E-21	1.26E-20
MAP4K4	133.74	91.25	1.47	3.76E-23	2.66E-22
PRKCG	3.38	2.29	1.48	1.74E-02	2.40E-02
HRAS	18.52	12.37	1.50	3.52E-12	1.15E-11
HSPB1	60.24	40.01	1.51	3.87E-12	1.24E-11
TGFBR1	106.44	70.13	1.52	6.14E-19	3.21E-18
STK3	41.26	26.65	1.55	1.64E-18	7.88E-18
HSPA6	5.49	3.43	1.60	3.67E-05	6.40E-05
ELK1	49.96	30.79	1.62	4.90E-24	3.81E-23
DUSP10	27.04	16.22	1.67	3.95E-14	1.59E-13
FGF1	4.77	2.79	1.71	9.30E-06	1.75E-05
TP53	105.07	59.63	1.76	3.25E-24	2.79E-23
HSPA1B	102.12	55.34	1.85	4.49E-24	3.61E-23
PDGFRB	166.24	87.76	1.89	8.03E-28	1.02E-26
TGFB2	9.23	4.52	2.04	8.04E-14	3.13E-13
FGF18	1.90	0.85	2.22	1.27E-05	2.36E-05
HSPA8	631.28	273.40	2.31	5.34E-56	1.29E-53
CACNG8	7.57	3.27	2.32	7.89E-10	2.19E-09
STMN1	77.81	31.07	2.50	1.15E-40	4.63E-39
DUSP4	56.69	21.44	2.64	2.53E-22	1.65E-21
CDC25B	169.88	60.96	2.79	9.71E-54	7.80E-52
IL1A	7.56	2.39	3.17	2.02E-19	1.08E-18
NTF4	0.55	0.17	3.23	3.80E-06	7.57E-06
CACNG4	3.41	1.03	3.30	8.11E-10	2.22E-09
MYC	181.11	49.00	3.70	5.76E-55	6.94E-53
FGF20	0.72	0.14	5.29	2.29E-05	4.15E-05
FGF19	1.39	0.15	9.42	6.08E-13	2.12E-12

Supplemental Table S3. Differentially Expressed genes in MAPK-Signaling Pathway in MSS-specific CRC tumors

Gene Name	Tumor Mean	Normal Mean	Fold Change	P-Value	Adjusted P-Value
FGF9	1.54	8.90	0.17	1.97E-28	3.17E-27
PPP3R2	0.21	0.93	0.23	4.56E-06	9.08E-06
CACNG3	0.14	0.59	0.23	5.85E-04	9.43E-04
CACNA1A	3.12	13.04	0.24	1.78E-31	3.90E-30
CACNA1F	1.61	6.05	0.27	3.75E-19	2.26E-18
CACNG7	0.14	0.53	0.27	9.99E-05	1.73E-04
CACNA1I	1.78	6.53	0.27	3.54E-18	1.90E-17
PRKCB	13.53	49.63	0.27	3.87E-37	1.87E-35
RASGRP2	6.50	23.81	0.27	3.67E-28	5.52E-27
CACNA1G	1.32	4.52	0.29	1.15E-12	4.34E-12
MAPK10	9.11	27.60	0.33	1.54E-31	3.70E-30
MAP4K1	7.00	20.62	0.34	8.27E-23	6.64E-22
CACNG2	0.21	0.60	0.36	4.12E-03	6.05E-03
FGFR2	23.56	63.63	0.37	1.11E-20	7.62E-20
IL1R2	6.10	15.74	0.39	1.10E-16	5.41E-16
NTRK1	0.95	2.40	0.39	2.24E-08	5.63E-08
PRKACB	63.18	158.66	0.40	1.47E-38	8.88E-37
FOS	185.07	459.66	0.40	2.51E-27	3.36E-26
MEF2C	26.21	63.01	0.42	2.02E-36	8.12E-35
CACNA1B	1.68	3.97	0.42	2.63E-06	5.36E-06
DUSP1	67.85	158.64	0.43	4.90E-31	9.83E-30
PTPN5	0.96	2.17	0.44	3.19E-06	6.41E-06
CACNB2	26.70	60.56	0.44	9.67E-35	2.91E-33
CACNG1	0.29	0.65	0.45	3.18E-04	5.35E-04
FGF10	1.75	3.85	0.45	8.70E-08	2.12E-07
NTRK2	18.31	38.56	0.47	1.84E-07	4.30E-07
DUSP5	28.22	58.81	0.48	2.43E-19	1.50E-18
NFATC1	10.80	22.32	0.48	6.38E-19	3.58E-18
CACNA1H	55.23	110.48	0.50	4.12E-24	4.52E-23
EGF	3.21	6.36	0.50	9.71E-04	1.50E-03
MAPT	4.55	8.91	0.51	1.45E-11	5.10E-11
FGFR3	37.26	72.10	0.52	1.35E-16	6.49E-16
MAPK7	20.82	39.85	0.52	1.36E-27	1.92E-26
FAS	25.99	49.72	0.52	3.54E-23	3.16E-22
RASGRP1	19.70	37.52	0.53	7.47E-22	5.46E-21
FASLG	0.91	1.69	0.54	3.27E-04	5.47E-04
CACNA2D3	1.56	2.86	0.54	6.14E-04	9.68E-04
PDGFRA	90.72	165.26	0.55	4.37E-22	3.29E-21
RASGRP3	24.55	44.61	0.55	4.51E-19	2.59E-18
NTF3	0.76	1.37	0.55	3.90E-04	6.47E-04
FGF2	9.95	17.73	0.56	1.46E-11	5.10E-11
RAC2	20.46	36.16	0.57	6.93E-16	3.09E-15
FGF7	7.94	13.85	0.57	1.23E-08	3.20E-08
RPS6KA1	87.10	151.23	0.58	6.69E-31	1.24E-29
FLNC	47.73	80.92	0.59	2.52E-09	7.23E-09
FGF13	2.46	4.15	0.59	1.46E-04	2.52E-04
CACNA1S	0.60	1.00	0.59	2.39E-02	3.26E-02
RPS6KA5	18.00	29.77	0.60	2.32E-14	9.65E-14
PRKACG	0.20	0.33	0.61	1.39E-02	1.96E-02
JUND	129.44	213.08	0.61	5.03E-32	1.35E-30

ARRB1	52.57	86.10	0.61	4.31E-23	3.71E-22
CACNA2D2	10.31	16.68	0.62	3.49E-08	8.67E-08
HSPA2	7.42	11.54	0.64	1.37E-05	2.63E-05
NR4A1	211.96	329.74	0.64	1.16E-08	3.03E-08
MAP3K14	33.49	51.65	0.65	1.30E-23	1.25E-22
PLA2G4F	24.66	38.02	0.65	1.47E-08	3.76E-08
CACNB4	5.02	7.73	0.65	3.59E-05	6.46E-05
MKNK1	62.71	95.68	0.66	6.93E-24	6.96E-23
IL1R1	57.04	86.97	0.66	3.76E-14	1.54E-13
MAP3K6	19.42	29.43	0.66	4.05E-11	1.36E-10
MAP2K6	25.21	38.16	0.66	3.46E-09	9.69E-09
PTPN7	15.71	23.76	0.66	5.15E-07	1.15E-06
CD14	9.45	14.25	0.66	1.12E-07	2.68E-07
CACNA2D4	8.40	12.65	0.66	2.59E-07	5.89E-07
MAPK3	62.23	93.18	0.67	4.28E-17	2.15E-16
BDNF	4.78	6.93	0.69	5.46E-05	9.68E-05
RASGRP4	5.14	7.45	0.69	1.53E-05	2.90E-05
RAP1B	70.93	102.70	0.69	3.07E-23	2.84E-22
FGFR1	55.70	78.69	0.71	3.46E-09	9.69E-09
SOS2	90.85	127.09	0.71	4.17E-19	2.45E-18
TNFRSF1A	87.61	118.88	0.74	7.39E-23	6.14E-22
PDGFA	42.06	56.56	0.74	1.05E-08	2.82E-08
CACNA2D1	12.07	15.94	0.76	5.87E-04	9.43E-04
MAP3K11	100.38	131.79	0.76	4.36E-14	1.72E-13
RAP1A	55.44	71.37	0.78	1.38E-10	4.38E-10
RPS6KA6	11.45	14.73	0.78	1.58E-02	2.22E-02
AKT3	40.61	52.05	0.78	5.75E-05	1.01E-04
PLA2G4A	5.63	7.21	0.78	9.03E-02	1.15E-01
MKNK2	186.33	237.93	0.78	1.84E-12	6.81E-12
FGF12	1.02	1.29	0.79	2.35E-01	2.82E-01
PLA2G4E	1.30	1.65	0.79	1.45E-01	1.81E-01
MAP2K5	28.83	36.31	0.79	2.42E-06	4.99E-06
MECOM	154.96	194.95	0.79	1.08E-08	2.85E-08
EGFR	173.25	217.89	0.80	1.44E-13	5.49E-13
PPM1A	112.53	141.41	0.80	4.18E-12	1.53E-11
RPS6KA2	43.27	54.32	0.80	1.15E-05	2.28E-05
DUSP8	43.21	53.67	0.81	3.08E-06	6.25E-06
MAP3K12	48.15	59.73	0.81	7.03E-09	1.93E-08
PPP3CC	16.60	20.57	0.81	6.02E-04	9.61E-04
FLNB	770.33	951.62	0.81	2.49E-10	7.49E-10
CRK	91.47	110.78	0.83	9.81E-10	2.88E-09
PRKCA	69.76	84.27	0.83	1.07E-07	2.57E-07
PPM1B	77.88	94.08	0.83	7.78E-09	2.11E-08
MAP3K13	83.26	99.51	0.84	1.33E-07	3.15E-07
MAP3K3	40.27	47.72	0.84	6.03E-05	1.05E-04
PLA2G4C	5.97	7.03	0.85	1.03E-01	1.29E-01
TAOK3	106.39	125.16	0.85	5.38E-07	1.19E-06
TAOK2	80.60	93.88	0.86	9.60E-07	2.05E-06
RELB	24.69	28.67	0.86	3.26E-03	4.88E-03
TAB1	28.72	33.07	0.87	6.07E-04	9.62E-04
MAP2K4	30.62	35.14	0.87	1.02E-02	1.44E-02
RRAS2	21.51	24.67	0.87	2.75E-02	3.72E-02
CACNB1	14.55	16.59	0.88	5.19E-02	6.79E-02

RAF1	129.93	147.72	0.88	1.88E-07	4.36E-07
MAP3K5	64.53	73.00	0.88	9.98E-04	1.53E-03
PRKACA	53.66	60.68	0.88	3.21E-05	5.81E-05
TAB2	126.20	142.60	0.89	1.54E-05	2.90E-05
CACNA1E	5.79	6.53	0.89	3.68E-01	4.23E-01
KRAS	102.76	115.27	0.89	6.55E-03	9.39E-03
NFKB1	74.35	83.07	0.90	5.04E-04	8.26E-04
FGF17	0.83	0.93	0.90	4.99E-01	5.47E-01
NFKB2	43.48	48.47	0.90	2.37E-02	3.25E-02
MAX	68.38	76.20	0.90	1.56E-03	2.37E-03
MAP3K1	71.55	79.29	0.90	3.11E-03	4.68E-03
FGF11	3.57	3.93	0.91	3.87E-01	4.38E-01
MAPK8IP3	132.45	145.48	0.91	3.79E-03	5.60E-03
PLA2G4B	39.21	42.79	0.92	5.21E-02	6.79E-02
GNG12	144.21	157.33	0.92	2.38E-02	3.25E-02
GADD45G	2.84	3.09	0.92	4.65E-01	5.16E-01
CASP3	40.44	43.73	0.92	1.24E-01	1.55E-01
PTPRR	10.94	11.79	0.93	5.51E-01	5.99E-01
JMJD7-PLA2G4B	68.08	73.14	0.93	6.06E-02	7.84E-02
MAP2K2	71.00	76.18	0.93	4.26E-02	5.61E-02
PPP3CB	53.74	56.85	0.95	1.86E-01	2.26E-01
IKBKB	139.63	146.41	0.95	1.80E-01	2.21E-01
MAP2K3	68.75	71.73	0.96	3.59E-01	4.14E-01
RASA1	124.54	129.40	0.96	1.95E-01	2.35E-01
MAP2K7	50.10	51.76	0.97	3.49E-01	4.08E-01
TNF	1.94	2.00	0.97	8.43E-01	8.52E-01
HSPA1L	2.91	2.99	0.97	7.65E-01	8.02E-01
ECSIT	18.77	19.27	0.97	6.66E-01	7.07E-01
AKT1	149.80	153.53	0.98	3.57E-01	4.13E-01
SOS1	156.26	159.10	0.98	5.47E-01	5.97E-01
PPP3CA	76.38	77.67	0.98	6.86E-01	7.25E-01
BRAF	54.68	55.58	0.98	6.39E-01	6.85E-01
PLA2G4D	1.06	1.08	0.99	9.58E-01	9.62E-01
MAPKAPK2	125.02	125.70	0.99	8.45E-01	8.52E-01
MAPK11	6.45	6.44	1.00	9.87E-01	9.87E-01
NFATC3	159.07	157.77	1.01	7.57E-01	7.96E-01
CDC42	141.68	140.45	1.01	7.74E-01	8.05E-01
MAPK8	60.07	59.48	1.01	7.92E-01	8.16E-01
LAMTOR3	29.96	29.66	1.01	8.43E-01	8.52E-01
MAPK1	179.19	177.32	1.01	6.62E-01	7.06E-01
DUSP6	80.28	79.16	1.01	7.75E-01	8.05E-01
TGFB1	39.73	39.15	1.01	8.01E-01	8.21E-01
RASA2	81.11	79.45	1.02	5.70E-01	6.16E-01
MAP3K2	194.54	190.31	1.02	3.79E-01	4.33E-01
CACNB3	32.84	31.97	1.03	6.18E-01	6.65E-01
NLK	48.15	46.84	1.03	4.80E-01	5.31E-01
RASGRF1	2.69	2.61	1.03	8.39E-01	8.52E-01
TRAF6	37.66	36.47	1.03	4.13E-01	4.65E-01
MAPK9	64.79	62.72	1.03	3.81E-01	4.33E-01
MAPK12	45.66	44.16	1.03	4.61E-01	5.15E-01
FLNA	1101.68	1063.82	1.04	4.93E-01	5.42E-01
GRB2	115.12	110.69	1.04	1.71E-01	2.12E-01
JUN	229.02	218.97	1.05	3.18E-01	3.76E-01

MAP3K8	21.28	20.33	1.05	4.58E-01	5.13E-01
PPP5C	60.57	57.07	1.06	9.09E-02	1.15E-01
ARRB2	39.12	36.53	1.07	1.77E-01	2.18E-01
FGF23	0.46	0.42	1.08	7.78E-01	8.05E-01
MAP3K4	69.37	64.11	1.08	1.81E-02	2.51E-02
IL1B	20.45	18.71	1.09	3.09E-01	3.67E-01
ATF2	68.23	62.24	1.10	7.04E-03	1.00E-02
RAPGEF2	158.39	143.63	1.10	7.70E-04	1.21E-03
IKBKG	7.43	6.74	1.10	2.64E-01	3.15E-01
PAK1	113.64	102.80	1.11	1.94E-03	2.94E-03
MAPK13	67.23	60.24	1.12	5.25E-03	7.57E-03
RELA	83.08	74.02	1.12	1.40E-05	2.68E-05
SRF	68.94	61.02	1.13	4.17E-04	6.89E-04
DUSP3	94.46	83.40	1.13	5.38E-04	8.77E-04
MAPK14	143.24	125.84	1.14	2.41E-05	4.43E-05
ELK4	252.34	221.55	1.14	1.64E-05	3.04E-05
GADD45A	12.80	11.20	1.14	6.08E-02	7.84E-02
GADD45B	13.22	11.56	1.14	7.81E-02	1.00E-01
MAPK8IP1	7.95	6.93	1.15	1.28E-01	1.60E-01
MAP2K1	31.83	27.72	1.15	3.42E-03	5.09E-03
CHUK	42.41	36.69	1.16	4.17E-03	6.09E-03
AKT2	159.97	136.80	1.17	3.68E-07	8.28E-07
RPS6KA3	253.94	216.56	1.17	2.56E-05	4.68E-05
TGFB3	16.85	14.25	1.18	3.99E-02	5.28E-02
TGFBR2	207.95	175.84	1.18	6.56E-07	1.44E-06
MAP3K7	72.57	61.36	1.18	1.06E-06	2.23E-06
MAPK8IP2	2.20	1.85	1.19	1.89E-01	2.28E-01
FGF5	0.99	0.82	1.21	3.55E-01	4.13E-01
MAP4K2	18.92	15.61	1.21	4.87E-03	7.07E-03
HSPA1A	33.27	27.28	1.22	1.72E-02	2.40E-02
ATF4	128.67	104.89	1.23	3.56E-11	1.21E-10
DUSP16	113.18	91.93	1.23	4.38E-08	1.08E-07
CRKL	128.52	104.39	1.23	2.05E-11	7.06E-11
RRAS	20.43	16.58	1.23	2.38E-04	4.07E-04
DUSP2	8.49	6.87	1.24	2.80E-02	3.75E-02
CACNA1C	74.60	58.92	1.27	1.59E-05	2.97E-05
MAPKAPK5	61.30	48.19	1.27	1.94E-09	5.63E-09
FGF14	0.61	0.48	1.28	3.39E-01	3.99E-01
RPS6KA4	45.96	35.13	1.31	8.98E-10	2.67E-09
DAXX	35.84	27.39	1.31	4.69E-11	1.55E-10
DDIT3	10.78	8.23	1.31	2.46E-04	4.18E-04
RAC3	2.65	2.02	1.31	2.77E-02	3.73E-02
NF1	377.83	286.21	1.32	5.17E-24	5.42E-23
TAOK1	158.19	119.51	1.32	1.25E-19	7.91E-19
PDGFB	17.51	13.16	1.33	5.34E-05	9.52E-05
CACNA1D	94.38	70.52	1.34	6.98E-07	1.52E-06
DUSP7	23.34	17.09	1.37	1.83E-08	4.64E-08
RASGRF2	31.53	22.92	1.38	1.10E-06	2.32E-06
TRAF2	40.71	29.42	1.38	6.02E-12	2.17E-11
STK4	206.88	148.46	1.39	6.23E-16	2.83E-15
FGFR4	71.55	50.78	1.41	2.21E-10	6.73E-10
NRAS	105.16	74.25	1.42	1.73E-15	7.59E-15
HRAS	17.71	12.32	1.44	7.06E-09	1.93E-08

PAK2	186.50	129.57	1.44	3.44E-29	5.92E-28
PPP3R1	87.18	60.51	1.44	5.70E-18	2.99E-17
PRKCG	2.86	1.97	1.45	3.54E-02	4.72E-02
RAC1	185.06	126.22	1.47	2.06E-26	2.48E-25
MAP4K3	75.22	51.22	1.47	1.99E-16	9.42E-16
MRAS	14.22	9.65	1.47	1.39E-06	2.90E-06
MAPKAPK3	46.50	31.54	1.47	1.87E-14	7.89E-14
GNA12	74.01	50.07	1.48	1.07E-19	7.17E-19
STK3	38.46	25.93	1.48	3.92E-14	1.57E-13
HSPA6	5.27	3.51	1.50	7.94E-04	1.24E-03
MAP4K4	133.82	88.26	1.52	1.09E-26	1.38E-25
TGFBR1	103.38	67.72	1.53	4.94E-16	2.29E-15
HSPB1	60.73	39.72	1.53	5.13E-11	1.67E-10
DUSP10	25.70	16.54	1.55	1.38E-10	4.38E-10
ELK1	50.36	30.35	1.66	3.67E-22	2.85E-21
TP53	99.81	57.76	1.73	1.16E-19	7.58E-19
FGF1	4.96	2.80	1.77	1.17E-05	2.30E-05
HSPA1B	105.40	57.15	1.84	8.46E-21	6.00E-20
PDGFRB	172.44	87.63	1.97	2.69E-26	3.08E-25
TGFB2	9.19	4.09	2.25	3.22E-15	1.38E-14
HSPA8	614.77	265.35	2.32	2.92E-53	7.03E-51
DUSP4	45.68	19.40	2.35	1.24E-17	6.37E-17
CACNG8	6.93	2.84	2.45	1.62E-10	5.00E-10
STMN1	73.26	29.40	2.49	5.98E-36	2.06E-34
FGF18	2.01	0.76	2.65	2.17E-07	4.98E-07
CDC25B	169.79	59.15	2.87	3.20E-47	2.57E-45
IL1A	7.58	2.24	3.38	2.45E-18	1.34E-17
CACNG4	4.04	1.10	3.66	1.56E-10	4.89E-10
NTF4	0.62	0.16	3.79	8.54E-07	1.84E-06
MYC	192.41	49.64	3.88	2.13E-49	2.57E-47
FGF20	0.78	0.13	6.03	1.22E-05	2.37E-05
FGF19	1.64	0.13	12.27	1.19E-13	4.62E-13

Supplemental Table S4. Differentially Expressed Genes in MAPK-Signaling Pathway in MSI-Specific CRC Tumors

Gene Name	Tumor Mean	Normal Mean	Fold Change	P-Value	Adjusted P-Value
CACNA1G	0.96	7.40	0.13	1.49E-04	1.56E-03
RPS6KA6	3.23	15.91	0.20	2.65E-04	2.36E-03
PPP3R2	0.11	0.52	0.20	2.09E-02	5.44E-02
FGF10	1.12	5.22	0.22	3.27E-05	4.92E-04
EGF	1.33	5.68	0.23	6.17E-04	4.25E-03
FGF23	0.37	1.57	0.24	2.10E-02	5.44E-02
CACNG7	0.66	2.59	0.25	5.30E-02	1.11E-01
CACNG1	0.15	0.59	0.26	1.93E-03	1.06E-02
FGF9	2.79	10.17	0.27	1.06E-03	6.41E-03
CACNA1F	2.24	8.15	0.27	1.62E-03	9.07E-03
RASGRP2	8.71	31.49	0.28	5.40E-04	3.82E-03
CACNG3	0.42	1.52	0.28	1.75E-01	2.78E-01
PLA2G4E	0.59	1.97	0.30	8.86E-03	3.01E-02
PRKACB	51.14	170.04	0.30	1.63E-06	4.95E-05
MAPK10	10.30	32.15	0.32	3.94E-04	3.06E-03
PRKCB	25.53	79.68	0.32	5.68E-06	1.37E-04
PRKACG	0.09	0.28	0.32	1.26E-03	7.24E-03
PLA2G4F	10.75	33.03	0.33	6.88E-04	4.60E-03
MAP4K1	9.26	28.32	0.33	4.66E-05	6.24E-04
PTPN5	0.74	2.16	0.34	7.27E-02	1.41E-01
MEF2C	34.75	95.49	0.36	1.64E-06	4.95E-05
RASGRF1	1.73	4.46	0.39	8.37E-03	2.92E-02
CACNG2	0.27	0.71	0.39	2.03E-01	3.17E-01
FGF14	0.10	0.26	0.39	7.26E-02	1.41E-01
FGF18	1.04	2.57	0.41	1.47E-01	2.42E-01
CACNA1S	0.36	0.87	0.41	8.29E-02	1.59E-01
PDGFRA	72.03	170.72	0.42	2.63E-05	4.22E-04
NTRK2	15.87	37.03	0.43	3.64E-02	8.21E-02
DUSP5	29.37	66.01	0.44	1.62E-04	1.63E-03
FGFR3	28.18	62.99	0.45	7.30E-04	4.63E-03
CACNA1I	3.33	7.38	0.45	2.61E-02	6.36E-02
CACNA1A	4.81	10.24	0.47	1.84E-02	5.15E-02
PLA2G4C	2.97	6.31	0.47	1.65E-02	4.80E-02
MAPK8IP1	3.49	7.37	0.47	1.14E-02	3.71E-02
FGFR2	50.25	104.08	0.48	4.41E-04	3.32E-03
CACNA2D3	1.73	3.58	0.48	1.27E-01	2.15E-01
CACNB2	27.73	55.61	0.50	8.28E-05	9.07E-04
FGF13	2.41	4.81	0.50	1.17E-01	2.03E-01
FOS	205.26	406.32	0.51	1.13E-03	6.65E-03
CACNA1H	41.94	82.05	0.51	5.36E-04	3.82E-03
PDGFA	28.32	53.67	0.53	1.77E-04	1.69E-03
DUSP1	68.42	128.35	0.53	1.82E-04	1.69E-03
NR4A1	146.87	264.51	0.56	7.12E-04	4.63E-03
MAPT	4.07	7.19	0.57	1.33E-02	4.10E-02
RASGRP4	5.72	10.06	0.57	1.90E-02	5.26E-02
FGF20	0.21	0.36	0.57	5.95E-01	6.96E-01
IL1R1	59.97	104.13	0.58	3.68E-04	3.00E-03
MAP2K6	23.79	41.27	0.58	5.02E-03	2.05E-02
BDNF	4.88	8.44	0.58	3.64E-02	8.21E-02
FGF5	1.21	2.06	0.59	3.38E-01	4.70E-01

HSPA1L	1.68	2.86	0.59	8.39E-02	1.59E-01
TGFBR2	116.38	192.01	0.61	7.63E-06	1.53E-04
FGF2	10.70	17.36	0.62	5.27E-03	2.09E-02
JUND	120.45	195.17	0.62	2.29E-05	3.95E-04
CACNA2D2	10.62	16.93	0.63	1.11E-01	1.96E-01
GADD45G	2.45	3.90	0.63	1.09E-01	1.95E-01
PTPN7	21.73	34.22	0.63	3.24E-02	7.50E-02
HSPA1A	12.47	19.20	0.65	1.08E-01	1.94E-01
RASGRP3	33.14	50.67	0.65	5.43E-03	2.11E-02
CACNA2D4	10.32	15.62	0.66	9.76E-02	1.81E-01
FGFR1	46.60	67.87	0.69	6.53E-03	2.38E-02
RAP1A	63.20	91.99	0.69	3.27E-04	2.82E-03
FGF12	1.08	1.57	0.69	4.73E-01	5.91E-01
FGF19	0.16	0.23	0.69	5.49E-01	6.65E-01
MAP3K14	35.95	51.93	0.69	4.17E-03	1.83E-02
GADD45B	10.44	14.69	0.71	9.38E-02	1.75E-01
MECOM	144.62	203.21	0.71	2.25E-02	5.70E-02
MKNK1	70.02	98.03	0.71	4.08E-05	5.79E-04
SOS2	94.63	131.76	0.72	4.07E-03	1.81E-02
MAPK3	64.20	89.11	0.72	1.31E-02	4.10E-02
RAC2	34.34	47.65	0.72	3.04E-02	7.21E-02
HSPA2	6.54	9.05	0.72	2.13E-01	3.30E-01
FAS	44.41	61.21	0.73	3.67E-03	1.67E-02
CACNA1B	2.76	3.79	0.73	3.98E-01	5.36E-01
RPS6KA5	22.69	31.08	0.73	1.69E-02	4.85E-02
FGF7	8.70	11.78	0.74	2.20E-01	3.34E-01
MAP4K2	16.00	21.65	0.74	1.03E-01	1.88E-01
NFATC1	21.38	28.63	0.75	1.63E-01	2.66E-01
DUSP2	7.44	9.90	0.75	2.26E-01	3.37E-01
ARRB1	56.54	75.00	0.75	2.93E-02	7.07E-02
CACNG4	0.29	0.39	0.75	6.39E-01	7.30E-01
RRAS	14.08	18.58	0.76	5.35E-02	1.11E-01
TNF	2.01	2.65	0.76	4.33E-01	5.56E-01
MAP3K3	39.77	52.38	0.76	5.28E-03	2.09E-02
RASGRP1	38.77	50.87	0.76	5.71E-03	2.19E-02
MAPK7	23.12	30.07	0.77	6.21E-03	2.30E-02
DUSP16	69.34	89.76	0.77	2.06E-03	1.06E-02
MAP2K5	30.64	39.58	0.77	5.87E-03	2.21E-02
CACNB4	10.31	13.29	0.78	4.38E-01	5.56E-01
GNG12	145.10	185.53	0.78	1.04E-02	3.43E-02
MAPK13	47.20	60.09	0.79	1.03E-02	3.43E-02
PRKCA	64.36	81.82	0.79	5.29E-02	1.11E-01
CACNA1C	45.89	58.11	0.79	2.23E-01	3.34E-01
DUSP8	35.32	44.69	0.79	1.20E-01	2.06E-01
MAPK8IP3	114.91	144.38	0.80	3.43E-03	1.59E-02
JUN	132.30	166.08	0.80	6.57E-02	1.33E-01
MKNK2	174.51	218.84	0.80	2.05E-02	5.44E-02
STK4	143.21	179.37	0.80	3.11E-03	1.49E-02
MAP3K11	102.63	128.22	0.80	2.79E-03	1.40E-02
AKT3	44.94	56.10	0.80	1.11E-01	1.96E-01
MAP3K13	96.45	119.88	0.80	4.30E-03	1.83E-02
RPS6KA1	103.34	125.35	0.82	4.01E-02	8.86E-02
NTF3	1.59	1.92	0.83	7.00E-01	7.72E-01

PPP3CC	19.95	23.98	0.83	2.52E-01	3.68E-01
NTRK1	3.23	3.88	0.83	6.69E-01	7.53E-01
RPS6KA2	47.76	57.28	0.83	1.33E-01	2.22E-01
PLA2G4B	32.21	38.53	0.84	1.20E-01	2.06E-01
TAB2	145.87	174.26	0.84	1.47E-02	4.43E-02
CD14	10.50	12.53	0.84	3.73E-01	5.13E-01
IKBKB	120.32	143.21	0.84	2.52E-02	6.19E-02
PPM1A	143.37	170.27	0.84	2.43E-02	6.09E-02
CACNB3	25.34	29.87	0.85	2.22E-01	3.34E-01
RPS6KA3	216.86	253.05	0.86	3.52E-02	8.08E-02
PRKACA	53.19	61.49	0.86	4.61E-02	1.01E-01
RAP1B	83.08	95.88	0.87	4.99E-02	1.07E-01
TAOK3	124.49	143.40	0.87	9.22E-02	1.74E-01
MAP2K2	64.51	73.95	0.87	1.65E-01	2.66E-01
RRAS2	29.07	33.25	0.87	3.10E-01	4.34E-01
SOS1	179.60	204.67	0.88	1.18E-02	3.80E-02
RELB	26.30	29.93	0.88	2.05E-01	3.18E-01
MAP3K2	211.92	240.77	0.88	6.85E-02	1.36E-01
MAP2K7	48.78	54.69	0.89	9.89E-02	1.82E-01
EGFR	190.81	213.26	0.89	1.41E-01	2.35E-01
FLNB	877.22	967.82	0.91	2.88E-01	4.18E-01
JMJD7-PLA2G4B	57.41	63.27	0.91	3.72E-01	5.13E-01
CACNB1	16.47	18.04	0.91	6.03E-01	6.99E-01
IL1R2	12.48	13.65	0.91	7.35E-01	7.98E-01
GRB2	111.23	120.87	0.92	1.82E-01	2.87E-01
PPM1B	87.83	95.43	0.92	2.99E-01	4.27E-01
NLK	47.66	51.11	0.93	3.80E-01	5.19E-01
RASGRF2	31.74	33.67	0.94	7.14E-01	7.82E-01
MAP3K12	51.43	54.46	0.94	5.13E-01	6.35E-01
FLNC	44.34	46.63	0.95	7.85E-01	8.45E-01
TNFRSF1A	111.85	116.86	0.96	5.80E-01	6.88E-01
CACNA2D1	13.91	14.51	0.96	8.36E-01	8.79E-01
MAPK14	128.95	134.12	0.96	4.18E-01	5.45E-01
TAOK2	85.17	88.57	0.96	5.36E-01	6.52E-01
CACNA1D	71.23	73.98	0.96	7.82E-01	8.45E-01
RASA2	81.27	83.82	0.97	7.01E-01	7.72E-01
MAX	86.79	88.36	0.98	8.24E-01	8.74E-01
MAP3K1	92.80	94.41	0.98	8.01E-01	8.58E-01
ATF2	78.40	78.90	0.99	9.37E-01	9.61E-01
ECSIT	18.04	18.11	1.00	9.78E-01	9.94E-01
RAF1	143.83	143.94	1.00	9.89E-01	9.96E-01
DUSP3	82.73	82.76	1.00	9.94E-01	9.96E-01
NFATC3	165.66	165.72	1.00	9.96E-01	9.96E-01
LAMTOR3	35.75	35.66	1.00	9.82E-01	9.95E-01
MAPK9	74.49	74.05	1.01	9.36E-01	9.61E-01
TAB1	31.07	30.81	1.01	9.22E-01	9.57E-01
MAPKAPK2	122.04	120.48	1.01	8.53E-01	8.89E-01
RASA1	143.79	141.63	1.02	8.39E-01	8.79E-01
CACNA1E	9.44	9.25	1.02	9.47E-01	9.68E-01
PPP3CB	70.38	68.91	1.02	7.31E-01	7.97E-01
ELK4	286.78	277.98	1.03	6.26E-01	7.19E-01
MAPK8IP2	2.83	2.75	1.03	9.34E-01	9.61E-01
TRAF6	40.34	38.97	1.04	6.87E-01	7.67E-01

RAPGEF2	144.92	139.32	1.04	5.62E-01	6.74E-01
MAP3K4	72.29	69.48	1.04	6.25E-01	7.19E-01
MAP3K7	70.84	67.81	1.04	5.91E-01	6.96E-01
DAXX	34.38	32.89	1.05	6.55E-01	7.41E-01
ARRB2	42.91	40.93	1.05	6.99E-01	7.72E-01
MRAS	14.58	13.91	1.05	8.07E-01	8.61E-01
AKT2	136.94	130.43	1.05	4.35E-01	5.56E-01
TGFB2	8.90	8.43	1.06	8.27E-01	8.74E-01
MAPK8	75.77	71.01	1.07	3.89E-01	5.27E-01
CDC42	169.50	158.13	1.07	2.22E-01	3.34E-01
CRK	131.17	122.31	1.07	2.29E-01	3.38E-01
FGFR4	50.39	46.89	1.07	6.85E-01	7.67E-01
KRAS	142.77	132.38	1.08	3.09E-01	4.34E-01
BRAF	57.47	53.28	1.08	3.81E-01	5.19E-01
NFKB2	57.81	53.57	1.08	4.53E-01	5.72E-01
PPP5C	64.57	59.71	1.08	2.23E-01	3.34E-01
NRAS	100.87	93.26	1.08	2.99E-01	4.27E-01
SRF	67.27	61.98	1.09	4.03E-01	5.38E-01
MAPKAPK3	35.59	32.67	1.09	4.70E-01	5.90E-01
TGFB3	17.21	15.77	1.09	6.54E-01	7.41E-01
MAPK1	215.16	195.12	1.10	6.55E-02	1.33E-01
MAPK11	9.07	8.19	1.11	5.77E-01	6.88E-01
PTPRR	16.57	14.91	1.11	5.94E-01	6.96E-01
NF1	330.31	296.25	1.11	5.00E-02	1.07E-01
RELA	77.61	69.46	1.12	1.64E-01	2.66E-01
MAPK12	54.91	49.06	1.12	3.02E-01	4.28E-01
NFKB1	101.45	90.62	1.12	1.14E-01	1.99E-01
PDGFB	16.69	14.83	1.13	5.60E-01	6.74E-01
GADD45A	14.62	12.89	1.13	4.07E-01	5.39E-01
RAC1	152.46	134.03	1.14	7.65E-02	1.47E-01
DUSP6	90.61	79.34	1.14	2.92E-01	4.22E-01
TAOK1	158.61	137.70	1.15	3.82E-02	8.51E-02
MAP4K4	126.85	109.98	1.15	2.50E-01	3.67E-01
FLNA	911.82	785.16	1.16	1.72E-01	2.75E-01
CASP3	61.68	52.95	1.16	1.25E-01	2.12E-01
FGF11	7.70	6.28	1.23	4.36E-01	5.56E-01
MAP2K3	92.77	75.65	1.23	4.69E-02	1.02E-01
MAP2K4	40.36	32.83	1.23	2.46E-02	6.12E-02
MAP4K3	72.55	58.66	1.24	1.56E-02	4.65E-02
PAK2	188.08	152.03	1.24	1.32E-02	4.10E-02
IKBKG	4.38	3.53	1.24	5.30E-01	6.48E-01
TRAF2	37.45	30.19	1.24	6.14E-02	1.27E-01
AKT1	181.80	143.79	1.26	3.16E-03	1.49E-02
RPS6KA4	47.40	37.42	1.27	1.95E-02	5.34E-02
TGFB1	55.19	43.50	1.27	6.77E-02	1.36E-01
PPP3CA	117.24	92.35	1.27	2.15E-02	5.52E-02
MAP3K8	28.28	22.27	1.27	1.06E-01	1.92E-01
MAP3K5	96.59	75.74	1.28	3.05E-02	7.21E-02
GNA12	65.63	51.21	1.28	7.64E-03	2.75E-02
MAP3K6	32.58	25.29	1.29	7.11E-02	1.41E-01
FGF1	3.37	2.61	1.29	4.17E-01	5.45E-01
CHUK	58.66	45.28	1.30	8.65E-03	2.98E-02
MAP2K1	39.05	30.06	1.30	1.99E-02	5.39E-02

PAK1	126.76	97.11	1.31	9.31E-04	5.75E-03
ATF4	146.18	111.12	1.32	8.17E-03	2.90E-02
MAPKAPK5	53.65	40.11	1.34	1.63E-02	4.80E-02
HSPB1	57.54	42.49	1.35	3.08E-02	7.21E-02
FASLG	2.80	2.04	1.37	4.85E-01	6.03E-01
PLA2G4D	0.88	0.64	1.39	5.98E-01	6.96E-01
PRKCG	8.99	6.45	1.39	4.37E-01	5.56E-01
ELK1	47.43	33.83	1.40	4.40E-03	1.83E-02
CRKL	140.74	100.17	1.40	1.28E-06	4.95E-05
PDGFRB	124.27	88.24	1.41	1.37E-02	4.18E-02
NTF4	0.12	0.08	1.45	5.27E-01	6.48E-01
CACNG8	13.55	9.29	1.46	4.16E-01	5.45E-01
TGFBR1	128.74	87.37	1.47	3.73E-04	3.00E-03
PPP3R1	106.28	71.76	1.48	5.94E-07	4.77E-05
FGF17	1.62	1.07	1.52	4.04E-01	5.38E-01
DDIT3	12.31	8.04	1.53	2.09E-02	5.44E-02
DUSP7	27.70	17.68	1.57	2.88E-03	1.42E-02
RAC3	3.75	2.35	1.59	1.68E-01	2.70E-01
TP53	138.21	72.39	1.91	7.60E-07	4.77E-05
HRAS	24.90	12.98	1.92	6.66E-06	1.46E-04
HSPA1B	81.97	42.07	1.95	5.00E-05	6.34E-04
STK3	65.27	33.16	1.97	1.98E-05	3.67E-04
IL1B	40.36	19.60	2.06	4.36E-03	1.83E-02
IL1A	7.60	3.63	2.09	1.82E-02	5.15E-02
HSPA8	712.76	320.23	2.23	1.30E-06	4.95E-05
CDC25B	173.03	75.19	2.30	1.25E-07	3.02E-05
MYC	111.44	45.58	2.44	7.91E-07	4.77E-05
STMN1	112.01	45.77	2.45	2.18E-06	5.83E-05
PLA2G4A	18.70	6.93	2.70	2.07E-03	1.06E-02
DUSP10	38.03	13.84	2.75	5.46E-05	6.57E-04
HSPA6	6.42	2.29	2.81	2.03E-03	1.06E-02
DUSP4	168.39	44.58	3.78	6.75E-05	7.75E-04
